# Integrative Analyses Identify a cGAS‐STING Pathway‐Driven Signature With Context‐Dependent Roles in Systemic Lupus Erythematosus

**DOI:** 10.1002/advs.202521560

**Published:** 2026-03-03

**Authors:** Lele Zhang, Ming‐Ju Amy Lyu, Ze Hong, Jing Sun, Lan Guo, Jiameng Hu, Munire Maimaiti, Chenhui Li, Jinyi Song, Xiang Zhang, Huansha Yu, Chen Wang, Xuan Wang, Haiyang Hu

**Affiliations:** ^1^ Central Laboratory Innovation and Incubation Center Shanghai Pulmonary Hospital School of Medicine Tongji University Shanghai China; ^2^ State Key Laboratory for Plant Molecular Genetics Center of Excellence for Molecular Plant Sciences Institute of Plant Physiology and Ecology Chinese Academy of Sciences Shanghai China; ^3^ State Key Laboratory of Technologies for Chinese Medicine Pharmaceutical Process Control and Intelligent Manufacture Nanjing University of Chinese Medicine Nanjing China; ^4^ State Key Laboratory of Natural Medicines School of Life Science and Technology China Pharmaceutical University Nanjing China; ^5^ Department of Rheumatology and Immunology Tongji Hospital School of Medicine Tongji University Shanghai China

**Keywords:** pristine‐induced lupus‐like mice, *Sting* ^−/−^ mice, systemic lupus erythematosus, the cGAS‐STING pathway, *Trex1* ^−/−^ mice, *Zbp1* ^−/−^ mice

## Abstract

The cGAS‐STING pathway is emerging as an essential driver in systemic lupus erythematosus (SLE). Here, we characterize the key signature of cGAS‐STING pathway and its roles in SLE by leveraging large‐scale transcriptomics, cell‐based assays, and two lupus‐like mouse models. We identify a STING‐dependent gene signature termed M7core, enabling quantitative assessment of cGAS‐STING pathway activity in SLE. M7core reveals widespread cGAS‐STING pathway activation in 70.4% of 3,180 SLE samples and predicts therapeutic response to STING antagonists in 74.1% of patients, with higher activity indicating greater sensitivity. Across ten independent cohorts, M7core outperforms interferon‐stimulated gene signatures (mean AUROC = 0.876) and correlates with disease activity, anti‐dsDNA antibodies, lymphopenia, and lupus nephritis. Hydroxychloroquine treatment reduces M7core expression and its clinical associations. Importantly, in cGAS‐STING pathway‐driven lupus‐like mice, STING antagonist administration ameliorates multiorgan pathology and suppresses M7core genes participating in promoting inflammation, type I interferon, and cell death, including ZBP1—an established cGAS‐STING pathway facilitator. Notably, ZBP1 deficiency phenocopies blocking cGAS‐STING pathway‐mediated autoimmune pathology exacerbation in pristane‐induced lupus‐like mice, underscoring its context‐dependent roles in lupus pathogenesis. Together, these findings define M7core as a robust diagnostic and mechanistic biomarker and highlight the necessity of assessing pathway activity before initiating STING‐targeted therapy in SLE.

## Graphical abstract

Zhang et al. identify M7core, a critical cGAS‐STING pathway‐driven gene signature that is activated in most lupus patients’ blood and links to lupus disease severity, lymphopenia, and lupus nephritis. They further reveal the diagnostic and pathogenic characteristics of M7core and emphasize the importance of assessing pathway activity before initiating STING antagonist therapy in lupus.

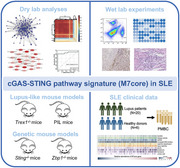

## 1 Introduction

Systemic lupus erythematosus (SLE) is a multifaceted autoimmune disorder characterized by a range of clinical manifestations, including pathogenic autoantibodies production, systemic inflammation, lymphopenia, and multiorgan damage [1]. SLE is highly heterogenous, and its exact cause remains unknown. Consequently, aside from two FDA‐approved targeted therapies, standard SLE treatment primarily involves nonspecific immunosuppression, often associated with debilitating side effects [2, 3, 4]. A hallmark of SLE is the production of antibodies to autologous double‐stranded DNA (dsDNA) [5]. These antibodies serve as diagnostic and prognostic markers and play essential roles in lupus pathogenesis, particularly in lupus nephritis (LN). Self‐dsDNA contributes to the initiation and progression of lupus through multiple mechanisms. Under normal physiological conditions, self‐dsDNA is confined to the nucleus and mitochondria, and any released harmful self‐dsDNA is rapidly degraded by cytoplasmic DNases, such as DNA exonuclease TREX1. However, in lupus patients, dysregulated cell death programs, including apoptosis, necroptosis, and NETosis, and/or defective DNA clearance, lead to the accumulation of self‐dsDNA. This triggers the production of anti‐dsDNA antibodies, the formation of immune complex (IC), activation of innate and adaptive immune responses, and multiorgan inflammation and destruction, eventually culminating in severe autoimmune manifestations of SLE [5, 6, 7, 8, 9, 10, 11].

Anti‐dsDNA antibodies are detected in individuals who developed SLE several years before the onset of symptoms and diagnosis and display the highest predictive odds ratio for predicting SLE disease [5, 7, 12], indicating that the cellular programs recognizing the dsDNA are critical to the onset and development of SLE. As the most prominent innate‐immune pathway highly conserved among mammalian species for dsDNA molecule sensing, the Cyclic GMP‐AMP Synthase (cGAS)—Stimulator of Interferon Genes (STING) pathway is emerging as one of the vital pathogenic drivers of lupus [13, 14, 15]. Upon dsDNA binding, cGAS is activated and catalyzes the production of the critical secondary messenger called cyclic GMP‐AMP (cGAMP) that binds to and activates STING. Activated STING initiates the downstream signaling cascade leading to the whole transcriptome changes, including but not limited to induction of IRF3‐dependent type‐I interferons, pro‐inflammatory cytokines, as well as recently identified several IRF3‐independent innate immune programs, all of which are orchestrated together for creating an efficient innate immune response environment [16, 17, 18, 19, 20, 21]. Accumulating evidence suggested that the cGAS‐STING pathway is engaged in SLE pathogenesis. A notable proportion of SLE patients display elevated serum cGAMP levels [22], and abundant dsDNA in apoptosis‐derived membrane vesicles enhances type I interferon production in a STING‐ or cGAS‐dependent, but TLR9‐independent, manner [23]. Furthermore, oxidized mtDNA derived from neutrophil extracellular traps (NETs) stimulates STING‐dependent type‐I interferon signaling in SLE [24]. STING autoactivation induced by gain‐of‐function mutants is also associated with certain lupus‐like manifestations in humans [25]. Strikingly, 69.2% of a cohort of 26 SLE patients have defects in the programmed mitochondrial removal during erythropoiesis, causing the accumulation of mitochondria‐containing red blood cells, the engulfing of which by macrophages activate the cGAS‐STING pathway‐dependent inflammation [26]. In addition, exposure to ultraviolet light, which is known to trigger and exacerbate SLE syndromes, led to cell death and induced type‐I interferon signature exclusively through the cGAS–STING pathway with a facilitation of ZBP1 in mice models [27, 28, 29]. Of note, loss‐of‐function mutations in the essential DNA exonuclease enzyme TREX1 cause cytosolic dsDNA accumulation, resulting in monogenic SLE in a proportion of patients and lupus‐like manifestations in *Trex1* ^−/−^ mice [30, 31]. Importantly, the depletion of cGAS or STING rescues *Trex1* ^−/−^ mice from mortality and autoimmune/autoinflammation manifestations, demonstrating that the lupus‐like phenotype in *Trex*1^−/−^ mice is primarily driven by the cGAS‐STING pathway [32]. Intriguingly, in pristane‐induced lupus‐like mice with autoimmune manifestations driven predominantly by Toll‐like receptor 7 (TLR7), blocking the cGAS‐STING pathway exacerbates autoimmune pathology with poorly understood mechanisms [33, 34]. Although these observations strongly demonstrated a close association between the cGAS‐STING pathway and SLE pathogenesis, the key molecular signatures and functional roles of this pathway in SLE remain incompletely understood. Moreover, the prevalence of cGAS‐STING activation and its clinical correlations across multiple SLE patient cohorts remains largely unknown.

In this study, we investigated these questions through an integrative approach by leveraging large‐scale in‐house and public transcriptomes, cell‐based assays, and two lupus‐like mouse models. Through systematic integration of time‐course transcriptomics upon cGAS‐STING pathway activation, network‐based module analysis, large‐scale transcription factor ChIP‐Seq data, and cell‐based assays from Bone Marrow‐Derived Macrophages (BMDMs) of *Trex1* ^−/−^ lupus‐like mice, we singled out a cGAS‐STING‐pathway‐driven gene signature, termed M7core (the core gene set of Module 7). M7core expression was STING‐dependent and modulated by both canonical and noncanonical signaling of the cGAS‐STING pathway. Notably, M7core activity perfectly correlated with cGAMP stimulation levels, enabling quantitative assessment of cGAS‐STING pathway activity. Based on M7core, we found that cGAS‐STING pathway was broadly activated in 70.4% of 3,180 SLE patients’ blood samples. We further demonstrated that pharmacological inhibition of the cGAS‐STING pathway using the STING antagonist significantly repressed M7core in 74.1% of 27 SLE patients’ peripheral blood mononuclear cells (PBMCs), with those showing higher M7core activity being more sensitive to STING inhibition, thus substantiating a broad activation of the cGAS‐STING pathway in SLE. M7core outperformed classical ISG (interferon‐stimulated gene) signatures in distinguishing SLE patients from healthy controls, with an average AUROC of 0.876 across ten independent cohorts. By leveraging more than 3,400 public RNA‐Seq profiles of 25 immune cells of PBMCs from SLE patients and healthy controls, we further showed the remarkable induction of M7core in all major immune cell types of SLE patient's PMBCs and its excellent diagnostic power for both myeloid cell types and lymphocyte types. M7core correlated strongly with key SLE clinical features, including SLE disease activity, dsDNA binding, lymphopenia, and lupus nephritis. Further examination of the transcriptome, histopathology, and immunohistochemistry of multiorgan in cGAS‐STING pathway‐driven lupus‐like mice after STING antagonist administration revealed a close association between M7core and disease manifestations. Importantly, in cGAS‐STING pathway‐driven lupus‐like mice, systemic tissue inflammation as well as M7core genes with established roles in promoting inflammation, cell death, and type‐I interferon, including ZBP1, were all significantly repressed after STING antagonist administration in multiorgan, indicating pathogenic roles of M7core genes. Of note, in pristane‐induced lupus‐like mice that is predominantly driven by TLR7 signaling, deficiency of the M7core gene ZBP1 recapitulated autoimmune pathology exacerbation observed upon cGAS‐STING pathway blockade, probably through more enhanced activation of TLR7 signaling downstream effectors p38 and STAT1, underscoring its context‐dependent role in lupus pathogenesis and the importance of patient stratification based on pathway activity for the effective use of STING‐targeted therapies in SLE.

## 2 Results

### 2.1 Integrative Analysis Identifies M7core as a Key Signature Driven by the cGAS‐STING Pathway

**FIGURE 1:**
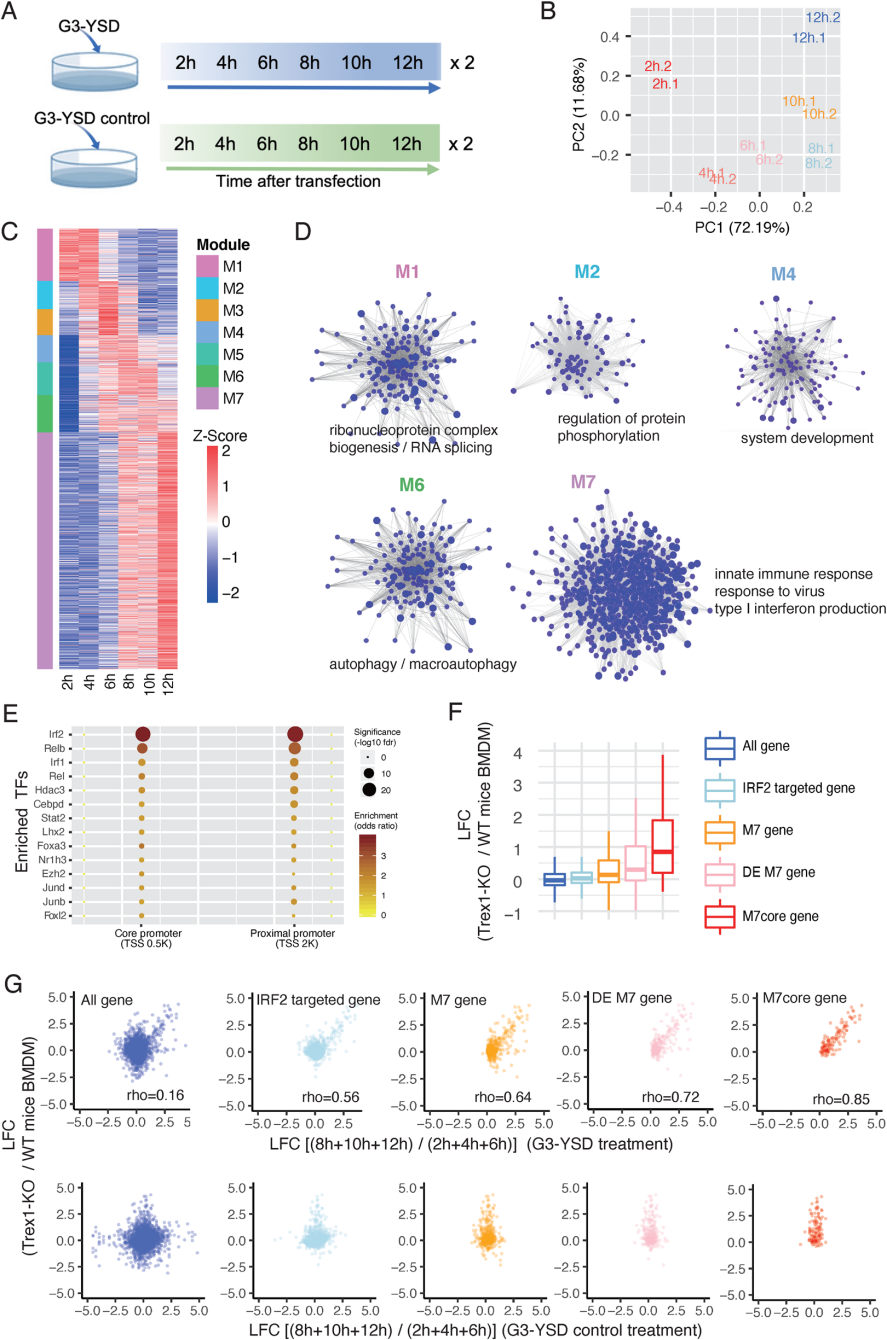
Integrative transcriptome analysis identifies M7core as a key cGAS‐STING pathway‐driven signature. (A) The schematic experiment design of time‐course RNA‐Seq after stimulating the cGAS‐STING pathway using cGAS agonist G3‐YSD and corresponding G3‐YSD control sequence (*n* = 2, independent experiments). (B) The PCA analysis of 14 293 genes across the time course after the cGAS‐STING pathway activation. (C) The heatmap of expression pattern of seven gene regulatory modules (M1 to M7) of the cGAS‐STING pathway based on time‐course RNA‐Seq profiling. (D) The regulatory network and enriched biological processes of the identified modules of the cGAS‐STING pathway. (E) The enriched transcription factors bound on the core and proximal promoter regions of M7 genes based on large‐scale transcription factor ChIP‐Seq data of ENCODE. (F) The expression changes of M7core genes and the other four groups of genes (all expressed genes, IRF2 targeted genes, M7 genes, and differentially expressed M7 genes) in the BMDMs between *Trex1* ^−/−^ mice and WT mice. (G) The correlation between expression changes in the BMDMs of *Trex1* ^−/−^ mice and expression changes after G3‐YSD stimulation in MEFs for M7core genes and the other four groups of genes (upper row). The correlation between expression changes in the BMDMs of *Trex1* ^−/−^ mice and expression changes after G3‐YSD control sequence stimulation in MEFs for M7core genes and the other four groups of genes (bottom row).

To identify critical gene programs of the cGAS‐STING pathway underlying SLE, we first investigated the modular gene network upon cGAS‐STING pathway activation. To this end, we conducted time‐course RNA‐Seq experiments by activating the cGAS‐STING pathway in mouse embryonic fibroblasts (MEFs) using the cGAS‐specific agonist G3‐YSD (Figure 1A). In parallel, transfection with a control sequence of G3‐YSD was performed to assess nonspecific effects of the transfection procedure. A total of 14 293 genes were quantified (Table S1). Principal component analysis showed that the first two principal components (PC) accounted for up to 83% of total expression variance (Figure 1B), demonstrating a marked effect of the cGAS‐STING pathway activation on gene expression dynamics over time. By integrating the results of gene expression regression analysis, Markov clustering, and protein‐protein interaction information, we identified seven gene modules regulated by the cGAS‐STING pathway activation, each exhibiting distinct expression patterns and biological functions (Figure 1C,D; Tables S2 and S3). Of note, known biological processes regulated by the cGAS‐STING pathway, such as autophagy, response to virus, and type I interferon production, were readily rediscovered, supporting the validity of the experiments and gene module analysis performed (Figure 1D; Table S3).

Loss‐of‐function mutations in the DNA exonuclease TREX1 activate the cGAS‐STING pathway and cause monogenic lupus in a subset of patients [10]. As the cGAS‐STING pathway is fully responsible for mortality and lupus‐like manifestations in *Trex1* ^−/−^ lupus‐like mice model [32], this model is considered as a cGAS‐STING‐driven lupus‐like mouse model. We then explored which gene modules of the cGAS‐STING pathway were associated with lupus by examining their gene expression changes in *Trex1* ^−/−^ lupus‐like mice (Table S4). Overall, we observed consistent expression change patterns across seven modules in BMDMs from *Trex1* ^−/−^ mice compared to wild‐type (WT) mice, mirroring those seen in MEFs following G3‐YSD stimulation (Figure S1A). Similar patterns were also observed in the hearts of *Trex1* ^−/−^ mice (Figure S2A). Notably, module 7 (M7) showed the strongest induction in both BMDMs and hearts of *Trex1* ^−/−^ mice, suggesting a closer association between M7 and autoimmunity. Consistently, M7 genes were strongly enriched in innate immune‐related functions (Table S3). We thus focused on M7 for further analysis. Analysis of the large‐scale transcription factor (TF) ChIP‐Seq data of GTRD [35] revealed IRF2 as the top‐enriched transcription factor binding both core and proximal promoters of M7 genes (Figure 1E; Fisher's exact test, BH‐adjusted *p* < 6.96e‐26, odds ratio = 3.97 for the core promoter; BH‐adjusted *p* < 1.51e‐26, odds ratio = 3.73 for the proximal promoter). Intriguingly, IRF2 was also an M7 gene and bound to its own promoter, suggesting an intrinsic autoregulatory loop. Notably, by intersecting the IRF2‐targeted M7 genes and M7 genes that were significantly induced after G3‐YSD stimulation (Negative binomial test, FDR<0.05), we obtained a subset of M7 genes that was induced much higher compared with all M7 genes or differentially upregulated M7 genes (DE M7 gene) in *Trex1* ^−/−^ mice, which was referred to as M7core (Figure 1F; Figure S2B and Table S5). M7core expression changes in BMDMs and hearts of *Trex1* ^−/−^ mice also correlated more strongly with those observed after G3‐YSD stimulation than all M7 genes or DE M7 genes (Figure 1G; Figure S2C). By contrast, these patterns were not observed in the G3‐YSD control group (Figure 1G; Figure S2C). Notably, M7core genes showed substantially higher induction than known ISGs or IRF2‐targeted ISGs in both BMDMs and hearts of *Trex1* ^−/−^ mice (Figures S1B and S2D), demonstrating their distinction from conventional ISGs. Collectively, our analysis identified M7core as a distinct gene module of the cGAS‐STING pathway, showing the strongest induction in the cGAS‐STING‐driven lupus‐like mouse model.

### 2.2 M7core is STING‐Dependent and Serves as a Quantitative Indicator of cGAS‐STING Pathway Activity

**FIGURE 2:**
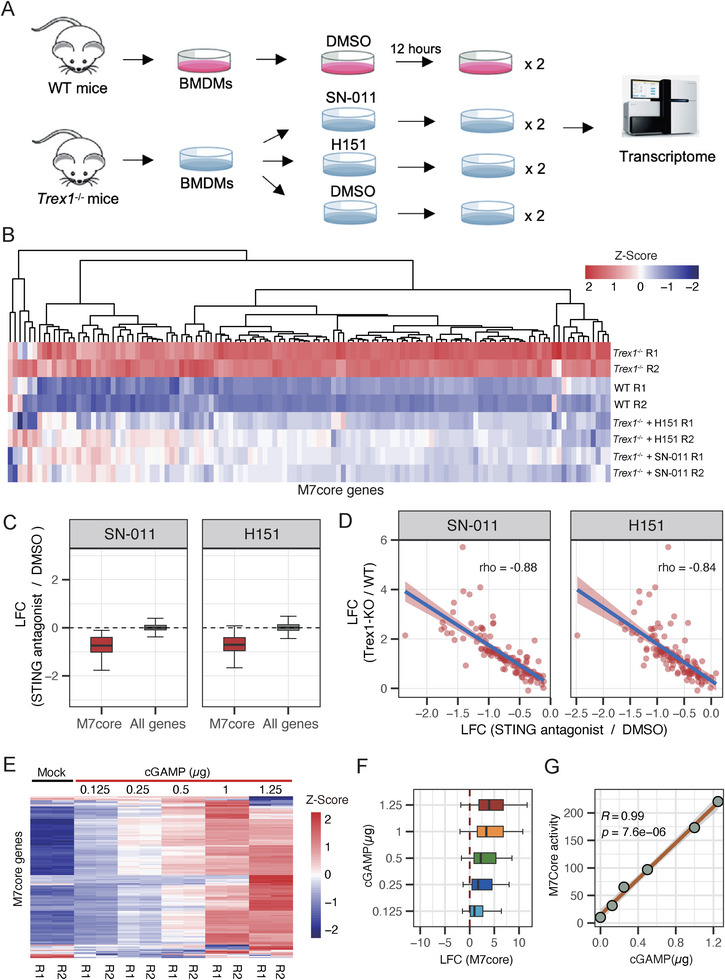
M7core is STING‐dependent and serves as a quantitative indicator of cGAS‐STING pathway activity. (A) Scheme of the experiment design for treating BMDMs of *Trex1* ^−/−^ mice with STING antagonists (SN‐011 or H151) or DMSO and for treating BMDMs of wild‐type (WT) mice with DMSO. (B) The heatmap of expression abundance of M7core genes in the WT and *Trex1* ^−/−^ BMDMs, with or without SN‐011/H‐151 treatment (*n* = 2, independent experiments). (C) The boxplot of expression changes of M7core genes and all expressed genes in the BMDMs of *Trex1* ^−/−^ mice after STING antagonist SN‐011 (left) and H151 (right) treatments. (D) The correlation between expression changes in the BMDMs of *Trex1* ^−/−^ mice and expression changes in the SN‐011 treated *Trex1* ^−/−^ BMDMs for M7core genes (left); the correlation between expression changes in the BMDMs of *Trex1* ^−/−^ mice and expression changes in the H151 treated *Trex1* ^−/−^ BMDMs for M7core genes (right). (E) The heatmap of expression abundance of M7core genes before (Mock) and after different levels of cGAMP stimulation (*n* = 2, independent experiments). (F) The boxplot of expression fold‐changes of M7core genes in log2‐scale (LFC) between Mock and cGAMP stimulations. (G) The correlation between M7core score and levels of cGAMP simulation.

To confirm the induction of M7core in *Trex1* ^−/−^ mice depended on the cGAS‐STING pathway, we isolated BMDMs from *Trex1* ^−/−^ mice, treated them with two highly potent STING antagonists (SN‐011 and H151), and performed RNA‐Seq experiments (Figure 2A; Table S4). Both antagonists inhibit the cGAS‐STING pathway efficiently but in distinct mechanisms [36, 37]. As shown in Figure 2B, almost all M7core genes were significantly suppressed in *Trex*1^−/−^ BMDMs following SN‐011 or H151 treatment (Negative binomial test, FDR < 0.05). Compared to all expressed genes, M7core activity was specifically suppressed (Wilcoxon rank sum test, *p* < 2.2e‐16 for H151, *p* < 2.2e‐16 for SN‐011; Figure 2C). Moreover, the magnitude of M7core gene repression following SN‐011 or H151 treatment was strongly anti‐correlated with their induction levels in *Trex1* ^−/−^ BMDMs (Figure 2D). In addition, reanalysis of public transcriptome data [37, 38] showed that M7core was strongly induced by STING agonist CMA in both BMDMs and primary CD4^+^ T cells, whereas this induction was nearly abolished by STING knockout or treatment with the STING antagonist C‐187 (Figures S3 and S4). Importantly, we further confirmed that the strong induction of M7core activity in *Trex1^−/−^* mice was almost completely repressed in *Sting^−/−^ Trex1^−/−^* mice [39] (Figure S5). Together, these results strongly demonstrated the dependence of M7core activity on the cGAS‐STING pathway.

We further investigated whether the expression level of M7core genes could serve as a readout of cGAS‐STING pathway activity. To test this, we stimulated BMDMs with increasing concentrations of cGAMP and conducted RNA‐Seq experiments (Table S6). The results showed a stepwise upregulation of M7core genes in response to increasing cGAMP levels (Figure 2E,F). We quantified M7core activity using a “M7core score”, defined as the median expression level of M7core genes, and observed a nearly perfect correlation with cGAMP stimulation levels (Figure 2G; Pearson correlation coefficient = 0.99, *p* < 8e‐6). Since cGAMP is the direct ligand of STING that largely determines the activation magnitude of the cGAS‐STING pathway [40, 41], these results indicate that the abundance of M7core genes can serve as a quantitative proxy for assessing cGAS‐STING pathway activity.

### 2.3 M7core is Partially Regulated by Type I Interferon

Recent studies have revealed interferon‐independent roles of the cGAS‐STING pathway [17, 18, 20, 21, 42]. Of note, introducing a serine‐to‐alanine mutation at position 365 (S365A) in STING that blocked the activation of IRF3 and downstream type I IFN response [17] only partially repressed M7core expression in BMDMs and T cells (Figure S6). This suggested that the canonical type I IFN pathway is only partially responsible for regulating M7core expression, consistent with the differential induction observed between M7core and conventional ISGs in *Trex1* ^−/−^ mice (Figures S1B and S2D). Consistently, 74 % of M7core genes were not in the gene list of three established IFN modules (Table S5). Moreover, 38.9% of M7core genes were regulated by RelB, a key component of non‐canonical NF‐κB signaling, which ranked as the second most enriched transcription factor at M7core gene promoters (Table S5). Collectively, the above results demonstrated that both canonical and noncanonical signaling of the cGAS‐STING pathway contributed to the regulation of M7core expression.

### 2.4 M7core Displays Superior Diagnostic Ability Than Conventional ISGs in SLE

**FIGURE 3:**
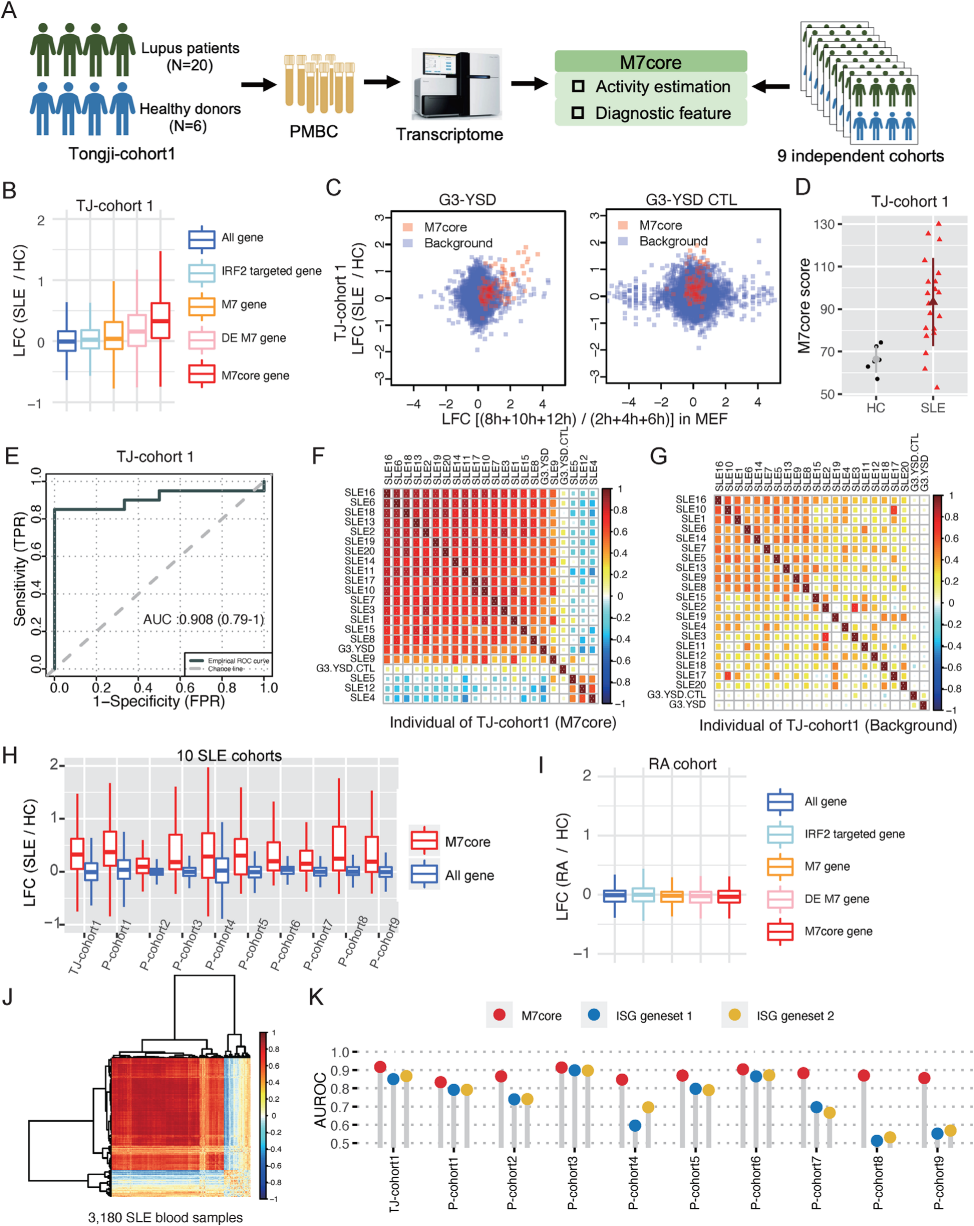
M7core displays superior diagnostic ability than conventional ISGs in SLE. (A) Scheme of the experiment design of Tongji‐cohort1 (*n* = 26). (B) The expression changes of M7core genes and the other four groups of genes (all expressed genes, IRF2 targeted genes, M7 genes, and differentially expressed M7 genes) in the PBMCs between SLE patients (SLE) and healthy donor controls (HC) in Tongji‐cohort 1(TJ‐cohort 1). (C) The correlation between expression changes in SLE patients and expression changes after G3‐YSD stimulation in MEFs for M7core genes (left); The correlation between expression changes in SLE patients and expression changes after G3‐YSD control sequence stimulation in MEFs for M7core genes (right). All expressed genes were used as the background (Background). (D) The comparison of the M7core score of each SLE and HC individual. (E) Receiver Operating Characteristic (ROC) curve and Area Under Receiver Operating Characteristic Curve (AUROC) value based on M7core genes in TJ‐cohort 1. (F) The pairwise correlation of expression changes of M7core genes in SLE patients, G3‐YSD treated MEFs, and G3‐YSD control sequence treated MEFs. (G) The pairwise correlation of expression changes of all expressed genes in SLE patients, G3‐YSD‐treated MEFs, and G3‐YSD control sequence‐treated MEFs. (H) The expression changes of M7core genes in the PBMCs or whole blood between SLE patients (SLE) and healthy donor controls (HC) in 10 independent SLE cohorts. (I) The expression changes of M7core genes and the other four groups of genes (all expressed genes, IRF2 targeted genes, M7 genes, and differentially expressed M7 genes) in the PBMCs between 112 RA patients (RA) and 45 healthy donor controls (HC). (J) The pairwise correlation of expression changes of M7core genes in 3180 PBMC or whole blood samples of SLE. (K) The comparison of AUROC value calculated based on M7core genes and ISG genes in 10 independent SLE cohorts.

Having established that M7core was dependent on the cGAS‐STING pathway and served as a quantitative indicator of its activity, we next examined M7core expression in SLE patients. We collected blood samples from twenty SLE patients and six healthy donors and performed RNA‐Seq experiments (Figure 3A, Tongji‐cohort 1 [TJ‐cohort 1], Table S7). Notably, M7core expression was significantly elevated in PBMCs from SLE patients compared to healthy donors (Wilcoxon rank sum test, *p* < 1.2e‐6; Figure 3B). Furthermore, M7core gene induction in SLE PBMCs significantly correlated with that in MEFs following G3‐YSD stimulation (Pearson correlation coefficient = 0.62, *p* < 1.515e‐12; Figure 3C). By contrast, no such correlation was observed following stimulation with the G3‐YSD control (Figure 3C). We further examined M7core activity in individual SLE patients and healthy donors using the M7core score. Most SLE patients exhibited higher M7core scores than healthy controls (Wilcoxon rank sum test, *p* < 0.0011; Figure 3D). Moreover, M7core genes distinguished SLE patients from healthy controls with an AUROC (Area Under the Receiver Operating Characteristic Curve) of 0.908 (Figure 3E). Notably, induction of M7core genes in 13 of 20 SLE patients was strongly correlated with that after G3‐YSD stimulation (Pearson correlation coefficient > 0.6; Figure 3F), distinguished from the pattern for all expressed genes (Figure 3G). Collectively, the above results indicated robust activation of M7core in SLE patient blood samples. To rule out cohort‐specific effects, we performed the same analysis across nine additional independent SLE cohorts comprising 3160 blood samples. Despite variability in age, sex, ethnicity, and technical platforms, M7core genes were consistently upregulated across all nine additional cohorts (Wilcoxon rank sum test, BH adjusted *p* < 0.01 for all SLE cohorts; Figure 3H), with induction levels significantly correlated with those of G3‐YSD stimulation in each SLE cohort (Figure S7). In contrast, M7core genes were not significantly induced in the blood of autoimmune disease rheumatoid arthritis (Wilcoxon rank sum test, *p* > 0.6; Figure 3I), suggesting the specificity of M7core to SLE. Importantly, pooled analysis of all 10 cohorts (*n* = 3180) revealed strong correlation between M7core activation and G3‐YSD‐induced gene expression in 2,239 samples (70.4%) (Pearson correlation coefficient > 0.6, *p* < 1.32e‐11; Figure 3J), confirming widespread M7core activation in SLE. Consistently, the M7core score demonstrated strong diagnostic power across all 10 cohorts, with an average AUROC of 0.876 (Figure 3K; Figure S8), significantly outperforming two conventional ISG gene sets (Wilcoxon rank‐sum test, *p* < 0.009; Figure 3K; Figures S9–S11).

By leveraging more than 3400 public RNA‐Seq profiles of 25 immune cells from PBMCs of SLE patients and healthy controls [43], we further found remarkable higher level of M7core activity in both myeloid cell types and lymphocyte types in SLE patients compared with healthy controls (Figure S12; Wilcoxon rank sum test, all FDR<1e‐10), indicating the broad activation of cGAS‐STING pathway in all major immune cell types of SLE patient's PMBCs. Evaluation of the diagnostic performance of M7core genes showed the robust and excellent diagnostic power for both myeloid cell types (Figure S13; AUC: 0.913‐0.943 across myeloid cell types) and lymphocyte types (Figures S14 and S15; AUC: 0.855‐0.951 across lymphocyte types) of purified immune subsets examined. Taken together, these results demonstrated broad activation of the cGAS‐STING pathway in SLE blood samples across 10 independent cohorts, and highlighted the strong diagnostic potential of M7core in distinguishing SLE from healthy controls.

### 2.5 STING Antagonist Significantly Represses M7core Activity in SLE

**FIGURE 4:**
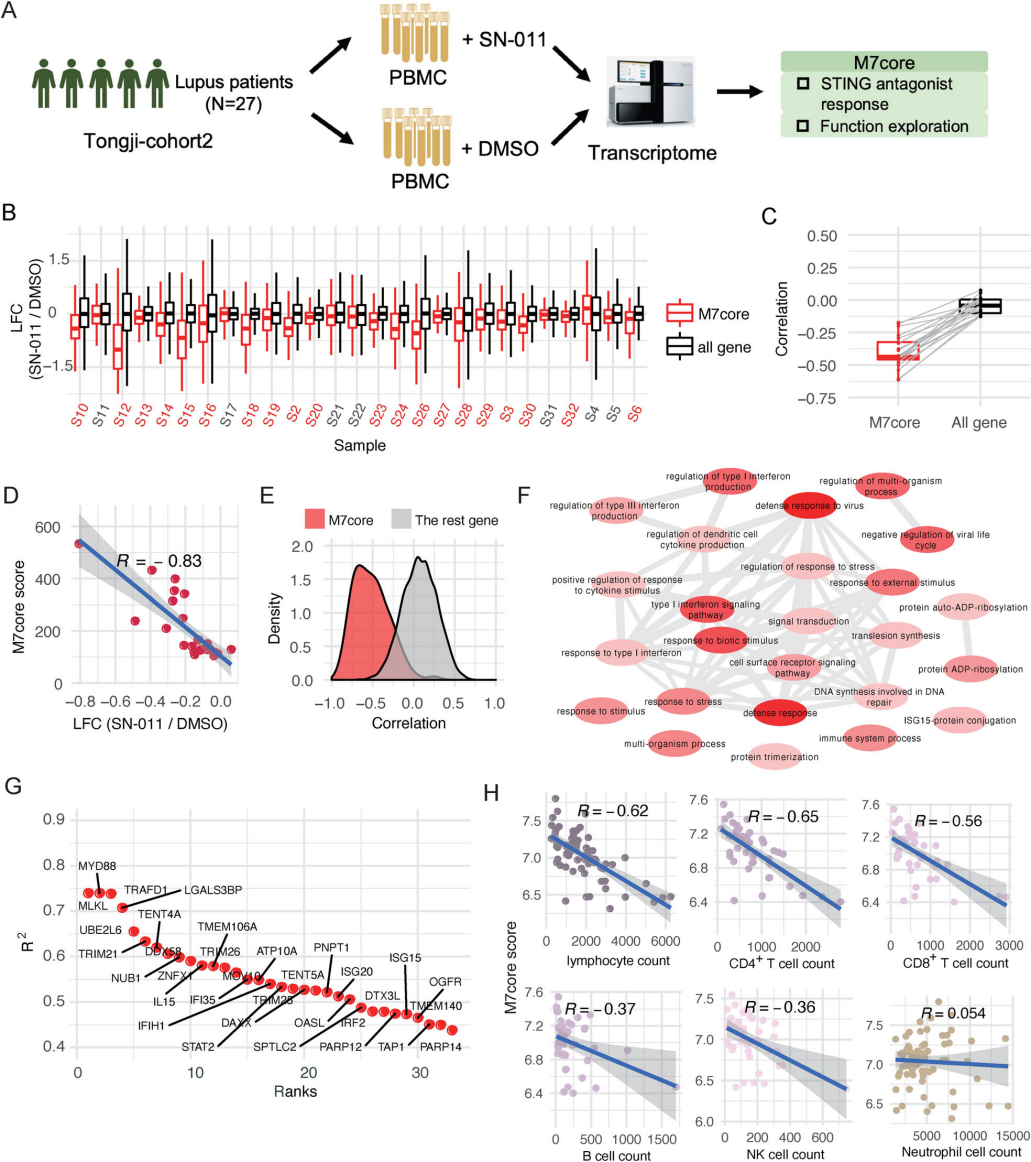
M7core is repressed in 74.1% of SLE patients after STING antagonist treatment and strongly associates with lymphopenia. (A) Scheme of the experiment design of Tongji‐cohort2 (*n* = 27). (B) The expression changes of M7core genes in the PBMCs after the inhibition of the cGAS‐STING pathway using STING antagonist. The expression changes of all expressed genes were used as controls. The SLE samples with significantly repressed M7core activity were labeled in red. (C) The boxplot of the correlations between the expression changes in STING antagonist‐treated PBMCs of SLE patients and the expression changes in G3‐YSD stimulated MEFs for M7core genes and all expressed genes. (D) The correlation between the activity of M7core estimated using the M7core score and the expression changes of M7core genes in the PBMCs after the inhibition of the cGAS‐STING pathway using STING antagonist. (E) The distribution of the correlation between the individual gene's expression abundance and corresponding expression changes in the PBMCs after the inhibition of the cGAS‐STING pathway using STING antagonist for M7core genes (red) and all the other genes (grey). (F) The enriched biological processes for the significantly downregulated M7core genes after STING antagonist treatment in SLE's PBMCs. The color shade was proportional to the enrichment significance. (G) The ranks of responsiveness to the STING antagonist were estimated using coefficient of determination (*R* ^2^) of the gene expression abundance and corresponding inhibition magnitude with SN‐011 treatments in the linear regression model. (H) The association between the M7core's activity and the counts of lymphocyte, CD4^+^ T cell, CD8^+^ *T* cell, B cell, NK cell, and Neutrophil cell in 158 blood samples of SLE patients.

To further confirm the broad activation of the cGAS‐STING pathway in SLE, we collected PBMCs from 27 SLE patients, treated them with the STING antagonist SN‐011, and performed RNA‐Seq, yielding 27 matched SN‐011‐ and DMSO‐treated transcriptomes (Figure 4A, Tongji‐cohort 2 [TJ‐cohort 2], Table S8). Notably, SN‐011 significantly suppressed M7core activity in 20 of 27 SLE PBMC samples (Wilcoxon rank sum test, BH adjusted *p*‐value < 0.05; Figure 4B and Table S9). Moreover, most of the 20 responsive patients showed a significant negative correlation between the degree of M7core inhibition by SN‐011 and its induction after G3‐YSD stimulation (Figure 4C; Table S10). Importantly, M7core activity and its inhibition magnitude by SN‐011 were strongly negatively correlated across all 27 patients (Pearson correlation coefficient = −0.83, *p* < 1.198e‐07; Figure 4D), indicating that patients with higher baseline M7core activity were more responsive to STING inhibition. Additionally, gene‐level analysis revealed that most M7core genes showed a negative correlation between expression levels and inhibition magnitude following SN‐011 treatment, exhibiting a distinct correlation pattern compared to background genes (Wilcoxon rank sum test, *p*‐value < 2.2e‐16; Figure 4E; Table S11). Collectively, these results substantiated the induction of M7core genes and the broad activation of the cGAS‐STING pathway in the blood of SLE patients.

### 2.6 M7core Genes MLKL and ZBP1 Correlate With Lymphopenia in SLE

We next explored the putative function of M7core genes in SLE. Functional enrichment analysis revealed that M7core genes significantly downregulated in SLE PBMCs following SN‐011 treatment were involved in innate immunity‐related processes, including defense response, regulation of type I interferon production, and interferon signaling, consistent with the known roles of the cGAS‐STING pathway (Figure 4F). To further dissect M7core genes, we ranked them by responsiveness to SN‐011 based on the coefficient of determination (*R* ^2^) between gene expression levels and inhibition magnitude using linear regression (Figure 4G; Table S11). Notably, the top ten M7core genes most responsive to STING inhibition included DDX58, an RNA sensor in the RIG‐I pathway, and MYD88, a receptor protein in IL‐1 and Toll‐like receptor pathways—both previously implicated in SLE [44, 45]. This result suggested a central role of the cGAS‐STING pathway in orchestrating other important pathogenic innate‐immune pathways in SLE. Intriguingly, MLKL, the terminal‐known obligate effector of cell necroptosis [46], was the most responsive M7core gene to STING inhibition (Figure 4G), suggesting that the cGAS‐STING pathway may mediate necroptosis in SLE PBMCs. Consistently, MLKL‐mediated necroptosis has been implicated in B‐cell lymphopenia in active SLE [47]. Moreover, ZBP1, a key regulator of PANoptosis and an essential facilitator of cGAS‐STING signaling [29, 48], was also among the M7core genes. Considering the role of cGAS‐STING pathway in promoting T cell apoptosis [49], we hypothesized that M7core‐mediated cell death programs may contribute to lymphopenia—a common feature in SLE with an estimated prevalence of 62% [50]. In line with our speculation, we observed strong negative correlations between M7core activity and counts of lymphocytes, CD4^+^ T cells, CD8^+^ T cells, B cells, and NK cells in 158 SLE blood samples (Figure 4H). No significant correlation was detected between M7core activity and neutrophil counts (Figure 4H). Hydroxychloroquine (HCQ), a first‐line therapy for SLE, has been reported to inhibit the cGAS‐STING pathway by blocking DNA sensing [51]. Notably, SLE patients not receiving HCQ exhibited a stronger association between M7core activity and lymphopenia, whereas this association was attenuated in those receiving HCQ (Figure S16A). Moreover, this observation persisted regardless of corticosteroid (CS) treatment (Figure S16A). Importantly, M7core genes were significantly downregulated following HCQ treatment in HT‐DNA‐stimulated PBMCs (Kolmogorov‐Smirnov test, *p* < 9.492e‐7; Figure S16B). These results provided evidence to support a critical role for M7core‐mediated cell death in contributing to lymphopenia in SLE. Notably, lymphopenia was significantly associated with expression of MLKL and ZBP1, but not with CD69—a known negative regulator of lymphocyte egress [52], suggesting that cell death, rather than impaired egress, may play a more dominant role in SLE‐associated lymphopenia (Figure S17).

### 2.7 Significant Clinical Relevance of M7core to Serologic Activity and Lupus Nephritis in SLE

**FIGURE 5:**
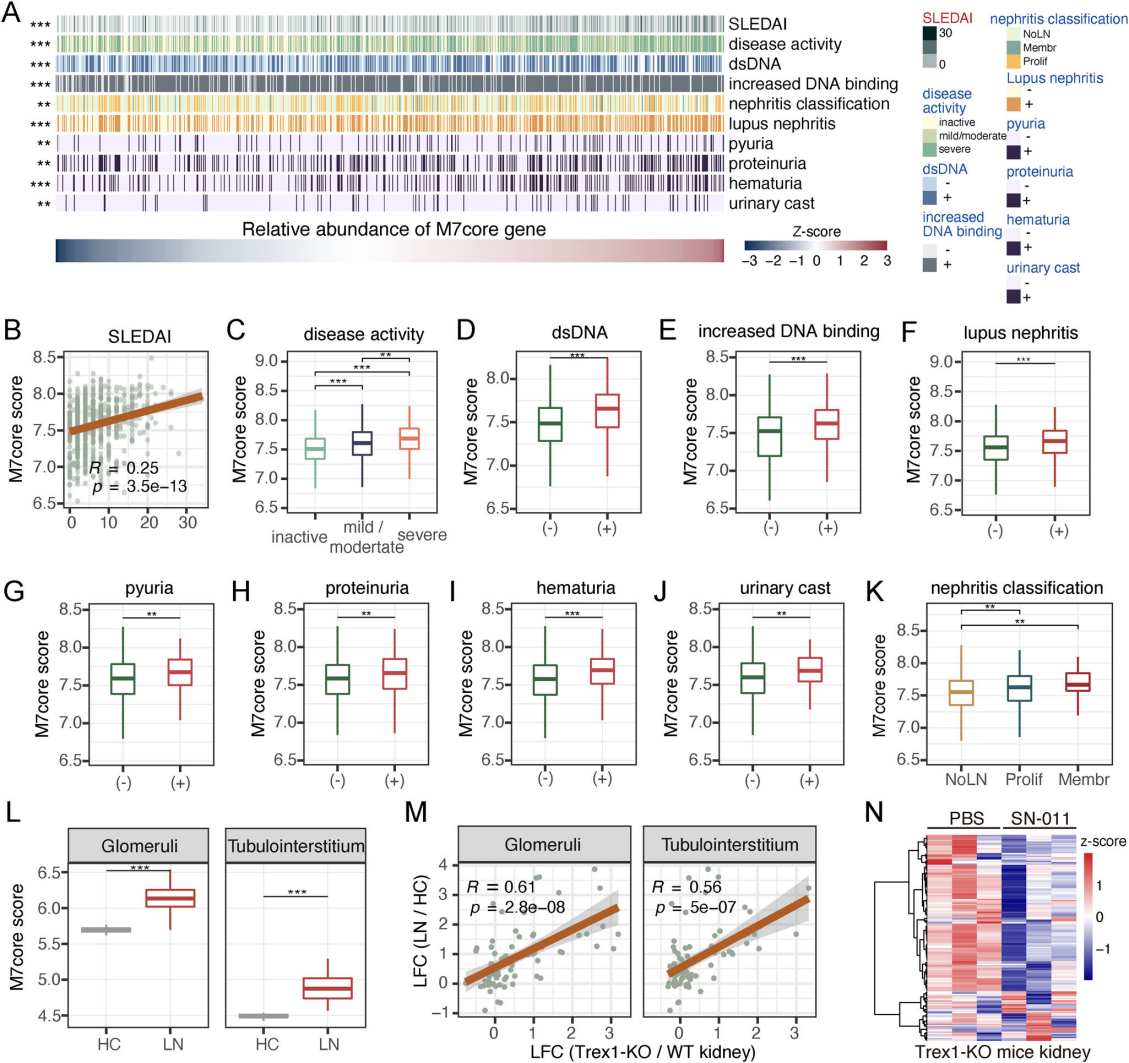
M7core correlates with serologic activity and lupus nephritis in SLE. (A) The summarization of clinical associations between the activity of M7core and serologic activity and renal‐related pathology in SLE. (B) The correlation between M7core's activity and SLE Disease Activity Index (SLEDAI). (C) The boxplot of M7core's activity in inactive (0–4), mild‐moderate (5–14), to severe disease activity status (≥15). (D) The boxplot of M7core's activity in anti‐dsDNA (+) and anti‐dsDNA (‐) SLE patients. (E) The boxplot of M7core's activity in SLE patients with and without increased dsDNA binding. (F) The boxplot of M7core's activity in SLE patients with (LN +) and without lupus nephritis (LN ‐). (G‐J) The boxplot of M7core's activity in SLE patients with and without pyuria, proteinuria, hematuria, and urinary casts, respectively. (K) The boxplot of M7core's activity in SLE patients with proliferative lupus nephritis (Prolif), Membranous lupus nephritis (Membr), and without lupus nephritis (NoLN). (L) The boxplot of M7core's activity in the glomeruli (left) and tubulointerstitium (right) of kidney biopsy in healthy control (HC) and SLE patients with lupus nephritis (LN). (M) The correlation between the expression changes of M7core genes in the glomeruli of kidney biopsy of SLE patients with lupus nephritis and the expression changes in the kidney of *Trex1* ^−/−^ mice (left); the correlation between the expression changes of M7core genes in the tubulointerstitium of kidney biopsy of SLE patients with lupus nephritis and the expression changes in the kidney of *Trex1* ^−/−^ mice (right). (N) The heatmap of the expression abundance of M7core genes in the kidney of *Trex1* ^−/−^ mice after two‐week STING antagonist SN‐011 administration (*n* = 3, per group). Statistical analysis was performed using one‐way ANOVA (disease activity and nephritis classification results of A), ANOVA followed by Tukey's test (C, K), two‐tailed Wilcoxon rank sum test for (dsDNA, increased dsDNA binding, lupus nephritis, pyuria, proteinuria, hematuria, and urinary cast results of A, D‐J, L), and correlation analysis (SLEDAI result of A, B, M). Statistical is represented by *p < 0.05, ** *p* < 0.01, ***p < 0.001.

The observed link between M7core and lymphopenia prompted a broader investigation into the clinical relevance of M7core in SLE. Using large‐scale gene expression data with matched clinical information from SLE blood samples [53], we detected significant clinical associations of M7core in SLE (Figure 5A). Specifically, M7core activity was significantly correlated with disease activity as measured by SLE Disease Activity Index (SLEDAI) (Pearson correlation coefficient = 0.25, *p* < 3.5e‐13; Figure 5B), showing stepwise increases across inactive (0‐4), mild‐to‐moderate (5‐14), and severe (≥15) disease states (Figure 5C; ANOVA followed by Tukey's test, *p* < 0.01). Intriguingly, the correlation between M7core activity and SLEDAI was stronger in patients without HCQ treatment (Pearson correlation coefficient = 0.45, *p* < 2.8e‐6; Figure S18A). By contrast, this correlation was markedly weaker in HCQ‐treated patients (Pearson correlation coefficient = 0.15, *p* < 1.5e‐5, Figure S18B). In addition, SLE patients with high anti‐dsDNA antibody titers exhibited significantly elevated M7core activity (Wilcoxon rank sum test, *p* < 3e‐8, Figure 5D). A similar result was observed for the SLE patients with increased DNA binding (Wilcoxon rank sum test, *p* < 6.2e‐5, Figure 5E). The high titer of anti‐dsDNA antibody is generally associated with lupus nephritis [6]. Consistently, M7core activity was significantly elevated in SLE patients with lupus nephritis (Wilcoxon rank sum test, *p* < 1.2e‐6, Figure 5F). SLE patients with pyuria, proteinuria, hematuria, or urinary casts all showed significantly higher M7core activity (Wilcoxon rank sum test, *p* < 0.0022 for pyuria, *p* < 0.0014 for proteinuria, *p* < 2.5e‐6 for hematuria, *p* < 0.0069 for urinary cast; Figure 5G–J). M7core activity was also higher in patients with proliferative or membranous lupus nephritis compared to those without nephritis (ANOVA followed by Tukey's test, *p* < 0.0032 for proliferative lupus nephritis, *p* < 0.0094 for membranous lupus nephritis; Figure 5K). The above results strongly demonstrated a link between M7core activity in blood and renal pathology in SLE. Additionally, M7core activity was significantly higher in both the glomeruli and tubulointerstitium of kidneys from SLE patients with lupus nephritis (Wilcoxon rank sum test, *p* < 2.6e‐12 for Glomeruli, *p* < 1.4e‐12 for Tubulointerstitium; Figure 5L). Similar results were observed in the infiltrating leukocytes in the kidneys of SLE patients with lupus nephritis based on single‐cell sequencing [54] (Figure S19). These results indicated that the cGAS‐STING pathway was activated in both the blood and the kidneys of SLE patients. To further investigate this association, we measured M7core activity in the kidney of *Trex1* ^−/−^ lupus‐like mice model using RNA‐Seq. M7core genes were significantly upregulated in the kidneys of *Trex1* ^−/−^ mice (Wilcoxon rank sum test, *p* < 2.1e‐7; Table S12). Importantly, M7core gene induction in the glomeruli and tubulointerstitium of SLE patients with lupus nephritis significantly correlated with that observed in *Trex1* ^−/−^mouse kidneys (Pearson correlation coefficient = 0.61, *p* < 2.8e‐8 for Glomeruli, Pearson correlation coefficient = 0.56, *p* < 5e‐7 in Tubulointerstitium; Figure 5M). To validate this, we treated *Trex1* ^−/−^ mice with the STING antagonist SN‐011 for two weeks and assessed M7core gene expression changes in the kidney using RNA‐Seq (Table S12). M7core expression was significantly suppressed in the kidneys of *Trex1* ^−/−^ mice following the cGAS‐STING pathway inhibition (Wilcoxon rank sum test, *p* < 0.024, Figure 5N). Collectively, these results demonstrated strong associations between M7core activity and both serologic activity and renal pathology in SLE.

### 2.8 M7core Involves in Multiorgan Pathology in cGAS‐STING Pathway‐Driven Lupus‐Like Mice

**FIGURE 6:**
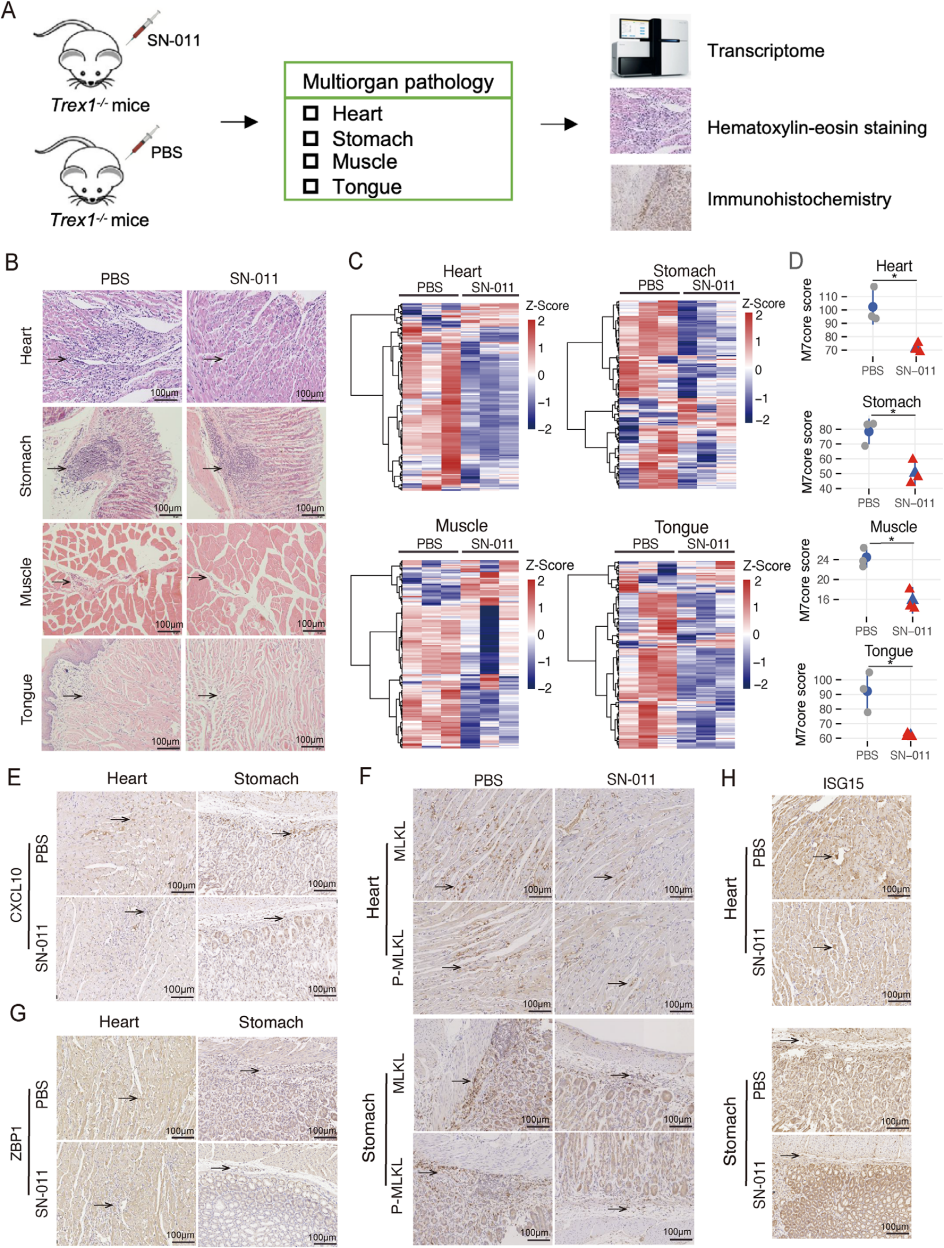
M7core associates with multiorgan pathology in the cGAS‐STING pathway‐driven lupus‐like mice model. (A) Scheme of the experiment design for measuring transcriptome and pathology changes across multiorgan after STING antagonist SN‐011 administration in the cGAS‐STING pathway‐driven lupus‐like mice model. (B) The representative Hematoxylin‐eosin (H&E) experiment results of multiple tissues (Heart, Stomach, Muscle, and Tongue) of *Trex1* ^−/−^ mice with two‐week administration of STING antagonist SN‐011 or PBS. Scale bar, 100 µm. (C) The heatmap of the expression abundance of M7core genes in the multiple tissues (Heart, Stomach, Muscle, and Tongue) of *Trex1* ^−/−^ mice with two‐week administration of STING antagonist SN‐11 or PBS (*n* = 3, per group). The corresponding heatmap result from the Kidney was shown in Figure 5N. (D) The comparison of M7core scores of the multiple tissues of *Trex1* ^−/−^ mice with two‐week administration of STING antagonist SN‐011 or PBS. (E‐H) The representative immunohistochemistry (IHC) staining of CXCL10 (E), MLKL and phosphorylated MLKL (F), ZBP1 (G), ISG15 (H) in the heart and stomach of *Trex1* ^−/−^ mice with two‐week administration of STING antagonist SN‐011 or PBS, respectively. Scale bar, 100 µm. Statistical analysis was performed using two‐tailed Wilcoxon rank sum test for (D). Statistical significance is represented by *p < 0.05.

Systemic inflammation and multiorgan damage are hallmarks of SLE pathology [55]. Based on the *Trex1* ^−/−^ lupus‐like mice model that was driven by the cGAS‐STING pathway activation, we explored the association between M7core activity and tissue pathology. To this end, we administrated *Trex1* ^−/−^ mice with STING antagonist SN‐011 intraperitoneally for two weeks and then examined the disease manifestation and M7core activity across multiple organs using Hematoxylin and Eosin (H&E) staining, immunohistochemistry (IHC), and RNA‐Seq experiments (Figure 6A; Table S12). The H&E staining and RNA‐Seq profiling showed that the cGAS‐STING pathway inhibition reduced immune cell infiltration and M7core gene expression across multiple affected tissues in *Trex1* ^−/−^ mice (Figure 6B–D; Figure S20A). Moreover, the M7core gene *Cxcl10*, a well‐known proinflammatory cytokine critical to lupus inflammation [56], was decreased at both mRNA and protein levels in multiple tissues (Figure 6E; Figure S20A). These results demonstrated a strong link between M7core activity and systemic inflammation in multiorgan of *Trex1* ^−/−^ mice. Cell death is a critical mediator of tissue inflammation and damage in lupus pathogenesis [8]. Notably, two M7core genes critical to cell death, *Mlkl* and *Zbp1*, were both downregulated in multi‐organ following SN‐011 administration, as shown by RNA‐Seq and IHC (Figure 6F,G; Figure S20A). Furthermore, MLKL phosphorylation was also reduced (Figure 6F). Given that phosphorylated MLKL executes necroptosis by inserting into the plasma membrane [46], these results suggest that STING inhibition alleviated necroptosis‐induced tissue damage via suppression of M7core genes *MLKL* and *ZBP1* in multiple tissues of *Trex1* ^−/−^ mice. Type I interferons are major pathogenic drivers in lupus. The M7core genes included known positive regulators of type I interferon signaling, including *ISG15* and *IFIT3* [57, 58]. RNA‐Seq and IHC analyses revealed reduced expression of *ISG15* and *IFIT3* following STING inhibition (Figure 6H; Figures S20A, S21), implicating M7core genes in type I interferon–mediated pathology across multiple organs in *Trex1* ^−/−^ mice. M7core gene expression changes were highly correlated across multiple affected tissues (Figure S20B), a pattern not observed for all expressed genes (Figure S20C). Together, these results indicated an involvement of M7core in multiorgan pathology in cGAS‐STING pathway‐driven lupus‐like mice.

### 2.9 Deficiency of M7core Gene ZBP1 Exacerbates Autoimmune Pathology in Pristane‐Induced Lupus‐Like Mice

**FIGURE 7:**
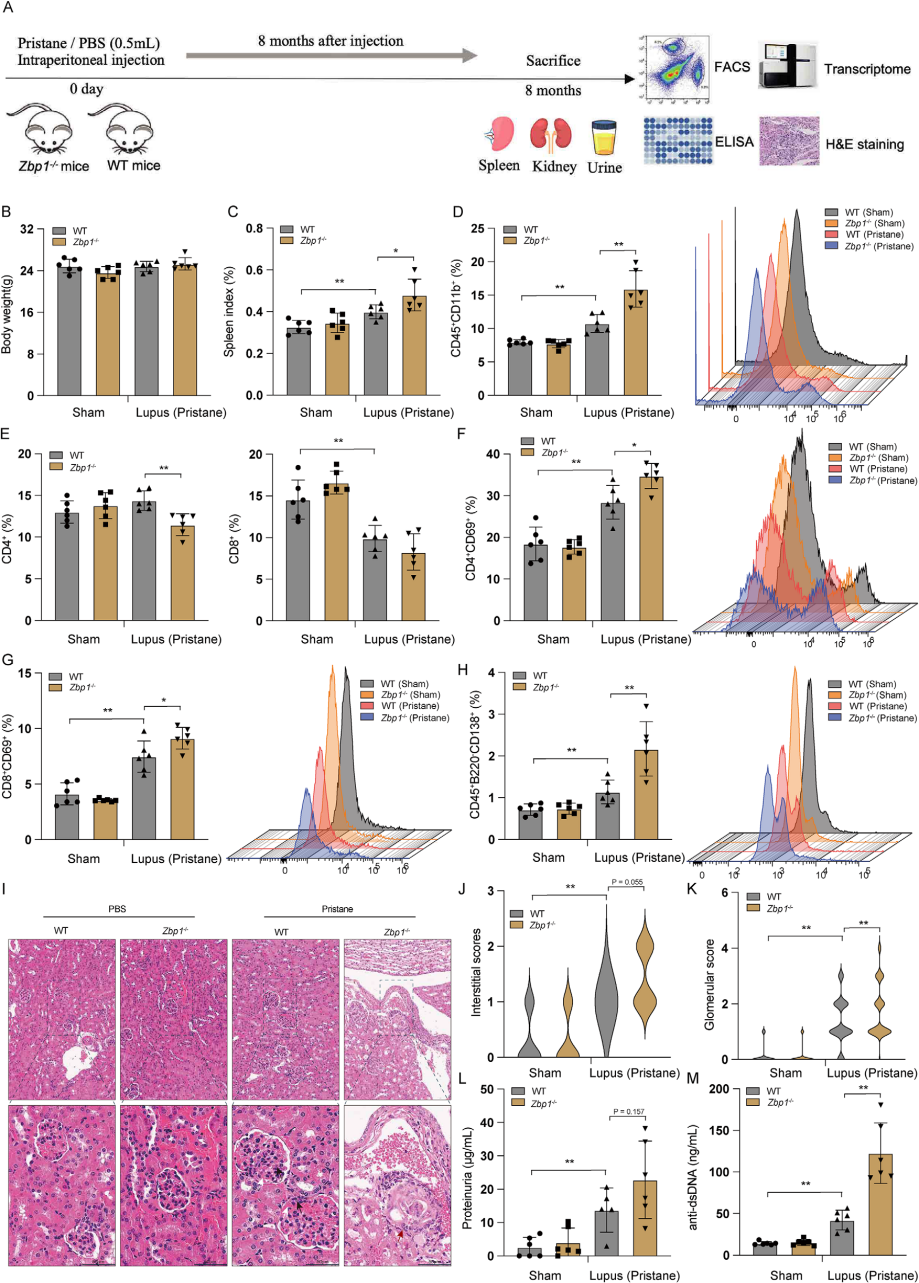
M7core gene ZBP1 deficiency exacerbates autoimmune pathology in the pristane‐induced lupus‐like mice. (A) Scheme of the experiment design for measuring autoimmune pathology in pristane‐induced lupus‐like mouse model. 8 weeks old female WT and *Zbp1* ^−/−^ mice were intraperitoneally injected with pristane or PBS, and autoimmune pathology was assessed 8 months post PBS or pristane administration. (B) Body weight was analyzed at 8 months post PBS or pristane administration (*n* = 6, per group). (C) Spleen index was calculated by the ratio of spleen weight to body weight of the experimental mice (*n* = 6, per group). (D) The percentage of CD11b^+^ cells among CD45^+^ cells in the spleens of the experimental mice (left), histogram shows representative overlays for CD11b signal (right) (*n* = 6, per group). (E) The percentage of CD4^+^ (left) and CD8^+^ (right) *T* cells in the spleens of the experimental mice (*n* = 6, per group). (F, G) The percentage of CD69^+^ cells (activated *T* cells) among CD4^+^ or CD8^+^ *T* cells in the spleens of the experimental mice (left), histogram shows representative overlays for the CD69 signal (right) (*n* = 6, per group). (H) The percentage of CD45^+^B220^−^CD138^+^ cells (plasma cells) in the spleens of the experimental mice (left), histogram shows representative overlays for CD138 signal (right) *(n* = 6, per group). (I) Representative H&E staining of kidney sections from WT and *Zbp1* ^−/−^ mice at 8 months after pristane injection. Infiltrated Neutrophil cells were marked with a black arrow, mesangial cell proliferation was marked with a white arrow, and glomerulosclerosis was marked with a blue arrow. Scale bar, 50 µm. (J, K) Interstitial (left) and glomerular (right) scores for renal pathology of the experimental mice. (L) Urine samples collected 8 months after pristane injection were tested for proteinuria using an albumin ELISA assay (*n* > 4, per group). (M) Anti‐dsDNA antibodies were measured in the serum at 8 months after pristane injection in WT and *Zbp1* ^−/−^ mice (*n* > 4, per group). All data in the statistical plots are shown as mean ± SD. Statistical analysis was performed using ANOVA followed by Tukey's test (B–H, J–M). Statistical significance is represented by *p < 0.05, **p < 0.01.

In the pristane‐induced lupus‐like mouse model with autoimmune manifestations driven predominantly by Toll‐like receptor 7 (TLR7), deficiency in STING or cGAS exacerbates disease pathology [33, 34]. Building on this model, we investigated the role of M7core gene ZBP1, a key facilitator of cGAS‐STING pathway, in modulating autoimmune pathology. To evaluate the effect of ZBP1 on pristane‐induced lupus pathogenesis, we generated ZBP1 deficiency (*Zbp1* ^−/−^) mice, intraperitoneally injected WT and *Zbp1* ^−/−^ mice with pristane for disease induction, and then assessed autoimmune pathology eight months post‐induction (Figure 7A). While body weight was comparable between WT and ZBP1 deficiency mice (Figure 7B), splenomegaly and increased myeloid cells (CD11b^+^) were observed following pristane injection, and these phenotypes were exacerbated in *Zbp1* ^−/−^ mice (Figure 7C,D). Lymphopenia characterized by a significant decrease of CD4^+^ and CD8^+^ T cells was presented in the spleen of pristane‐induced *Zbp1* ^−/−^ mice, whereas the lymphopenia in WT mice was milder, with CD4^+^ T cells remaining unchanged following pristane injection (Figure 7E and Figure S22A). Although the percentage of CD4^+^ and CD8^+^ T cells was reduced in the spleen of pristane‐induced mice, the ratio of their activated subsets (CD69^+^) was significantly increased in pristane‐injected mice, and ZBP1 deficiency further strengthened this tendency (Figure 7F,G). Although the proportion of pan‐B cells in the spleen was not significantly increased, pristane‐induced *Zbp1* ^−/−^ mice exhibited more mature plasma cells (B220^−^CD138^+^) specialized for sustained antibody production compared to WT mice (Figure 7H; Figure S22B,C). Histological analysis revealed the manifestations of lupus nephritis at eight months post‐pristane injection, and the renal pathology was aggravated in *Zbp1* ^−/−^ mice with scattered glomerulosclerosis (Figure 7I). Overall, pristane‐induced *Zbp1* ^−/−^ kidneys exhibited significantly increased interstitial thickening and glomerulonephritis after pristane induction (Figure 7J,K). RNA‐Seq analysis revealed differentially upregulated genes in pristane‐induced *Zbp1* ^−/−^ kidneys were strongly enriched in immune‐related processes, including innate immune responses and regulation of cytokines (Tables S13 and S14). Consistently, differentially upregulated ISGs were eight times more than differentially downregulated ISGs. Moreover, several cytokines were differentially upregulated (Figure S23). We further confirmed the significant expression elevation of innate immune (*Isg15*, *Oas2*) and inflammatory (*Tnf*) genes in *Zbp1* ^−/−^ kidneys compared to WT counterparts in pristane‐induced lupus‐like mouse using qPCR (Figure S22D). Additionally, urinary albumin assays revealed increased proteinuria in *Zbp1* ^−/−^ mice compared to WT counterparts after pristane injection (Figure 7L). We also quantified the production of anti‐dsDNA antibodies in the serum and observed significantly higher anti‐dsDNA antibodies content in pristane‐injected *Zbp1* ^−/−^ mice than in WT mice (Figure 7M). Collectively, these results indicated an exacerbated autoimmune pathology in the absence of ZBP1 in pristane‐induced lupus‐like mice.

We further explored the potential mechanisms underlying ZBP1 deficiency‐mediated autoimmune exacerbation. RNA‐Seq data analysis revealed no significant changes in the expression of M7core genes in *Zbp1* ^−/−^ mice compared to WT counterparts (Wilcoxon rank sum test, *p* > 0.4), suggesting that the cGAS‐STING pathway did not undergo substantial activation in the *Zbp1* ^−/−^ kidneys. This was further corroborated by Western blot results, which similarly showed no obvious activation of the cGAS‐STING pathway measured using phosphorylation levels of STING (p‐STING) and TBK1(p‐TBK1) in *Zbp1* ^−/−^ kidneys. Instead, we observed a significant increase of phosphorylation levels of p38 (p‐p38) and STAT1 (p‐STAT1), both of which are downstream signaling effectors of TLR7 signaling (Figure S24). While no significant changes were observed in the activation of JNK, another member of the MAPK family, the robust activation of p38 and STAT1 may contribute to the exacerbated immune response and autoimmune pathology observed in *Zbp1* ^−/−^ mice. The over‐activation of p38 and STAT1 of TLR7 signaling has been reported to play roles in enhanced T cell activation, increased mature plasma cells, elevated anti‐dsDNA antibodies, and more severe renal damage [59, 60, 61, 62], all of which were in line with the exacerbated autoimmune pathology in the *Zbp1* ^−/−^ kidneys of pristane‐induced lupus‐like mice (Figure 7). Taken together, these results demonstrated that deficiency of M7core gene ZBP1 exacerbated autoimmune pathology in pristane‐induced lupus‐like mice, possibly through more enhanced activation of TLR7 signaling. These observations implied the potential importance of ZBP1 in modulating immune signaling and maintaining immune homeostasis in the pristane‐induced lupus‐like model, and underscored its context‐dependent role in lupus pathogenesis.

## 3 Discussion

A hallmark of SLE is the production of autoantibodies that target the body's own cellular components, including self‐DNA. Beyond its role in pathogenic autoantibody production, self‐DNA can act as a damage‐associated molecular pattern (DAMP) when released from damaged or stressed cells, triggering immune recognition that leads to inflammation and tissue damage in SLE [5, 6]. Being the most prominent innate‐immune pathway for dsDNA sensing, the cGAS‐STING pathway has recently emerged as a vital pathogenic driver of SLE [13, 15]. Therefore, identifying key gene signatures of the cGAS‐STING pathway involved in SLE pathogenesis is essential for understanding its molecular mechanisms and developing targeted therapies. To this end, we conducted a comprehensive study integrating over 6000 gene expression profiles, cell‐based assays, and two lupus‐like mouse models. These integrative analyses enabled us to pinpoint a core gene module of the cGAS‐STING pathway in SLE, which we termed M7core. While serum cGAMP detection serves as an indicator of cGAS‐STING pathway activation, it is primarily qualitative, not quantitative, and lacks sensitivity. A similar situation is also true based on the measurement of STING phosphorylation level by Western Blotting. In contrast, assessing M7core activity through unbiased transcriptome data offered a quantitative method to evaluate cGAS‐STING pathway activation, enabling analysis of its prevalence, clinical associations, and pathogenic roles in SLE. Several intriguing observations emerged.

One notable finding is that, although SLE is a heterogeneous disease and the existence of a broad spectrum of age, sex, race, and data technical heterogeneity in different SLE cohorts, we detected a strong induction of M7core in 70.4% of 3,180 SLE samples across ten independent cohorts. Consistently, a classification model based on M7core expression demonstrated strong predictive power in distinguishing SLE from healthy controls, with an average AUROC of 0.876. Importantly, by generating the first transcriptome data from SLE PBMCs after pharmacological inhibition of the cGAS‐STING pathway using STING antagonist SN‐011, we found that 74.1% of SLE PBMCs responded to STING antagonist treatment, with higher M7core activity associated with greater susceptibility to STING inhibition. All these findings indicated a broad activation of cGAS‐STING pathway in SLE. This aligns with a recent report showing that 69.2% of 26 SLE patients exhibited cGAS‐STING pathway–dependent inflammation due to abnormal mitochondrial retention in red blood cells [26].

The identification of M7core also allowed us to uncover strong clinical associations of cGAS‐STING pathway in SLE pathogenesis, which has not been systemically investigated before. Based on M7core, we uncovered a strong association between the activity of cGAS‐STING pathway and the levels of anti‐dsDNA antibody and dsDNA binding. This finding aligns well with the known role of the cGAS‐STING pathway as a major DNA‐sensing innate immune mechanism, further supporting the validity of using M7core to estimate its activity. Importantly, M7core levels were significantly correlated with SLE disease activity, showing stepwise induction from inactive to mild‐moderate to severe states, indicating a key role of the cGAS‐STING pathway in SLE pathogenesis. Consistently, we observed a strong connection between cGAS‐STING pathway and lupus nephritis, a common complication affecting 40%–70% of SLE patients and leading to end‐stage renal failure within five years in approximately 10% [63]. Using M7core, we demonstrated that cGAS‐STING pathway activity was significantly elevated in SLE patients with proliferative or membranous lupus nephritis compared to those without nephritis and healthy controls. Moreover, STING antagonist strongly repressed M7core expression in the kidneys of *Trex1* ^−/−^ mice. Notably, it is known that lupus nephritis is more common in SLE patients who have high levels of autoantibodies, particularly those against dsDNA and those who have active disease [6]. These findings highlight the involvement of the cGAS‐STING pathway in SLE patients with lupus nephritis and support M7core as a reliable quantitative measure of its activation, offering a valuable tool for understanding its clinical relevance in SLE—particularly in relation to disease activity and nephritis development.

M7core includes MLKL and ZBP1, two key regulators of necroptosis, which were suppressed in STING antagonist‐treated SLE PBMCs and *Trex1* ^−/−^ mice, suggesting that activation of the cGAS‐STING pathway may promote necroptosis in SLE patients. Indeed, the cGAS‐STING pathway has recently been identified as a critical mediator of necroptosis, and MLKL‐driven necroptosis has been implicated in B‐cell lymphopenia in active SLE [47]. It is therefore intriguing to find that the activity of cGAS‐STING pathway gauged by M7core was remarkably inversely correlated with counts of lymphocytes, CD4^+^ T cells, CD8^+^ T cells, B cells, and NK cells in 158 blood samples of SLE patients, suggesting a pathogenic role of cGAS‐STING pathway in lymphopenia, a common manifestation of lupus patients with an estimated 62% prevalence. Interestingly, the proportion of SLE patients with activated cGAS‐STING pathway closely matched the prevalence of lymphopenia. Additional evidence supporting the involvement of the cGAS‐STING pathway in lymphopenia came from analysis of SLE patients receiving HCQ treatment. HCQ is a first‐line therapy for lupus. Recent studies have shown that HCQ inhibits the cGAS‐STING pathway by disrupting cGAS binding to cytosolic DNA [51]. Consistently, we showed that HCQ significantly reduced DNA‐induced cGAS‐STING pathway activation in human PBMCs. Importantly, the association between cGAS‐STING activity and lymphopenia was markedly reduced in HCQ‐treated patients, but remained strong in those not receiving HCQ, which was independent of corticosteroid treatment. These findings provided patient‐derived evidence implicating the cGAS‐STING pathway in lymphopenia. Notably, recent studies reported that the ZBP1‐MLKL necroptotic cascade induces cytoplasmic DNA accumulation, autonomously activating cGAS‐STING signaling [64]. Epidermal ZBP1 stabilizes mitochondrial Z‐DNA to drive UV‐induced IFN signaling in autoimmune photosensitivity via activating cGAS‐STING signaling [29]. Moreover, activation of STING and ZBP1 together promotes enhanced RIP3/MLKL phosphorylation and necroptosis [65]. Notably, a recent study found that MLKL activates the cGAS‐STING pathway by releasing mitochondrial DNA upon necroptosis induction [66]. It is therefore intriguing to speculate that cGAS‐STING pathway, ZBP1, and MLKL might form a positive feedback loop to drive the SLE pathogenesis.

As a quantitative indicator of cGAS‐STING pathway activity, M7core analysis across multiple organs in lupus‐like mice enabled tissue‐level assessment of pathway activation and pathogenic roles—an investigation not feasible in human lupus patients. Through pharmacological inhibition of the cGAS‐STING pathway in *Trex1* ^−/−^ lupus‐like mice with STING antagonist, we detected a substantial reduction of M7core's activity that closely correlated with the inflammation alleviation in multiple tissues. Additionally, several M7core genes known to promote tissue inflammation, cell death, and type I interferon induction were consistently downregulated across tissues in *Trex1* ^−/−^ lupus‐like mice. These results indicated the pathogenic roles of cGAS‐STING pathway in tissue inflammation and damage in SLE. In the *Trex1* ^−/−^ mice, the severity of renal pathological changes is known to be relatively modest compared with other affected organs, including the heart, stomach, muscle, and tongue. While STING inhibition significantly downregulated M7core activity in the kidney, the baseline histopathological alterations in *Trex1* ^−/−^ kidneys were considerably milder than those observed in these severely affected organs. As a result, conventional H&E staining was not sufficiently sensitive to robustly distinguish renal pathological differences between treatment groups. Importantly, despite the relatively subtle histological phenotype, M7core scoring revealed a clear and significant reduction of cGAS‐STING pathway activity in *Trex1* ^−/−^ kidneys upon STING inhibitor administration. This finding underscores a critical advantage of M7core with the ability to sensitively detect changes in cGAS‐STING pathway activity even in tissues where conventional histopathology provides limited resolution. We believe this sensitivity highlights the potential translational value of M7core for monitoring cGAS‐STING pathway activity and therapeutic response in future studies.

Another intriguing observation is that deficiency of the M7core gene ZBP1 exacerbated autoimmune pathology in the pristane‐induced lupus‐like mouse model. Previous studies have shown that inhibiting the cGAS‐STING pathway worsens autoimmune manifestations in the pristane‐induced lupus‐like model. The specific downstream genes of the cGAS‐STING pathway contributing to this phenotype remain incompletely understood. In this study, we identified the M7core gene ZBP1 as a contributor to exacerbated autoimmune pathology in the pristane‐induced lupus‐like mouse model. Although ZBP1 facilitates the cGAS‐STING pathway by promoting cytoplasmic DNA accumulation and stabilizing mitochondrial Z‐DNA to drive UV‐induced interferon signaling in autoimmune photosensitivity, its knockout significantly worsened autoimmune manifestations in the pristane‐induced lupus‐like mouse model, mirroring phenotypes seen in cGAS deficiency or STING deficiency pristane‐induced lupus‐like mice. These results indicate that ZBP1 serves as a critical effector of cGAS‐STING pathway in regulating autoimmune manifestations and underscore its context‐dependent roles in SLE pathogenies based on experimental lupus mice models. Although inhibitors targeting on the cGAS‐STING pathway have been developing by several large pharmaceutical companies and some promising results were obtained [67], our findings highlight the importance of assessing pathway activity before taking antagonist of cGAS‐STING pathway in SLE treatment. M7core may provide a means of identifying how to best deploy cGAS‐STING inhibitors in SLE.

IRF3 is a key transcription factor that transduces upstream cGAS‐STING pathway signals to induce type I interferons and interferon‐stimulated genes (ISGs), which have long been considered the primary output of the cGAS‐STING pathway. However, recent studies demonstrated unequivocally interferon‐independent roles of the cGAS‐STING pathway [17, 18, 20, 42]. We found that M7core genes extend beyond the downstream targets of type I interferons, as only 26% overlap with genes in three established IFN modules. In line with this, disrupting IRF3 activation and downstream type I IFN responses via the S365A STING mutation only partially suppressed M7core activity in BMDMs and T cells. Furthermore, M7core gene induction was significantly greater than that of ISGs following cGAS‐STING pathway activation. These results demonstrated that M7core genes comprised both interferon and interferon‐independent genes of the cGAS‐STING pathway. It should be noted that the M7core‐based classification model outperformed ISG‐based models in distinguishing SLE patients from healthy donors, suggesting that the interferon‐independent output of the cGAS‐STING pathway warrants greater attention in SLE research.

Recent studies have reported that individual genes or specific DNA methylation loci may serve as diagnostic or predictive biomarkers in SLE. While these single‐feature–based approaches can provide useful insights, they are often sensitive to cohort heterogeneity, technical variation, and disease stage. In contrast, transcriptome‐based multi‐gene signatures, particularly interferon‐stimulated gene (ISG) signatures, currently represent the most widely validated and robust gene expression‐based diagnostic tools for SLE across independent cohorts and platforms. For this reason, ISG signatures are generally considered the benchmark for evaluating new transcriptomic biomarkers in SLE. In this study, we therefore systematically benchmarked M7core against two representative ISG gene sets across ten independent SLE cohorts. This large‐scale comparison demonstrated that M7core consistently outperformed ISG signatures in diagnostic accuracy, as measured by AUROC, while also showing strong associations with disease activity, clinical manifestations, and therapeutic response. These findings suggest that M7core captures not only interferon‐driven transcriptional programs but also additional cGAS‐STING pathway‐specific outputs that are not fully reflected by ISG signatures alone. Together, this highlights the added value and robustness of M7core as a pathway‐informed transcriptomic biomarker for assessing cGAS‐STING pathway activity in SLE. From a translational perspective, the implementation of M7core as a clinical biomarker may not necessarily require comprehensive transcriptomic profiling. Given the strong internal coherence of the M7core that is a median‐based approach that could minimize the influence of outlier genes and platform‐specific dynamic range differences, it is conceivable that a reduced panel of representative genes may be sufficient to capture disease‐relevant cGAS‐STING pathway activity. Such an approach could facilitate the development of clinically practical assays based on PBMCs, using platforms that are already compatible with routine clinical workflows, including qPCR‐based panels or Nanostring technologies. Future studies will be needed to define the minimal gene set and analytical thresholds required for robust patient stratification, as well as to evaluate the clinical utility of M7core‐guided therapeutic decision‐making.

This study has several limitations. (1) Although cGAS‐STING pathway inhibition using a pharmaceutical approach significantly reduced immune cell infiltration and M7core gene expression across multiple affected tissues in *Trex1* ^−/−^ mice, and thus providing functional evidence that M7core activity is downstream of cGAS‐STING pathway in vivo, M7core was positioned as a biomarker reflecting cGAS–STING pathway activity, rather than definitive proof of a causal role in driving human SLE. (2) Although M7core exhibited strong correlations with disease activity, lymphopenia, and lupus nephritis, the evidence from human samples was largely associative. To establish a direct causal link between the cGAS‐STING pathway and the downstream M7core effector program in lupus, in vivo validation using a cell‐type‐specific genetic knockout model required further exploration. (3) Most analyses in this study are based on bulk PBMC and tissue transcriptomic data, which obscures the mechanistic link between cGAS‐STING pathway, lymphopenia, and tissue pathology. Future studies incorporating paired single‐cell transcriptomic data and lineage‐specific genetic knockout mice models are necessary to dissect the underlying cellular mechanisms in more detail.

## 4 Conclusion

In summary, our results uncover a critical gene signature of the cGAS‐STING pathway with both diagnostic and pathogenic relevance in SLE. M7core provides a quantitative measure of cGAS‐STING pathway activity, serving as a valuable tool for assessing its activation prevalence, clinical associations, and pathogenic roles in SLE. The broad activation of the cGAS‐STING pathway in multi‐cohorts of SLE patients, together with the promising results of STING antagonists in alleviating inflammation in the PBMCs of SLE patients and multiorgan of the cGAS‐STING pathway‐driven lupus‐like mice model, highlight the critical roles of cGAS‐STING pathway in lupus pathogenesis and suggest that blockage of the cGAS–STING pathway may represent a therapeutic strategy for treating lupus exhibiting an activated cGAS‐STING pathway. The finding that ZBP1 deficiency phenocopied the effects of blocking the cGAS‐STING pathway in exacerbating pathology in pristane‐induced lupus‐like mice underscores its context‐dependent role in lupus pathogenesis and highlights the importance of evaluating pathway activity before administering STING antagonists for lupus treatment.

## 5 Experimental Section

### 5.1 Patients

All SLE patients in the study met the American College of Rheumatology/European League Against Rheumatism (ACR/EULAR) 2021 criteria. All patients gave informed consent, and samples of peripheral blood mononuclear cells were collected. The committees of the Tongji Hospital (K‐2025‐109) and Shanghai Pulmonary Hospital (K25‐898) approved the study.

### 5.2 Mice

*Trex1* ^+/−^ mice were provided by Dr. Nan Yan from the University of Texas Southwestern Medical Center and kindly licensed by Dr. Tomas Lindahl and Dr. Deborah Barnes from Cancer Research U.K., London. *Trex1* ^−/−^ mice were obtained by genotyping the offspring after mating the male and female *Trex1* ^+/−^ mice. *Zbp1* ^+/−^ mice were generated with the CRISPR‐Cas9 technique by gempharmatech Co., Ltd (Strain NO. T029037). The exon 2‐exon 8 of Zbp1 gene was deleted to generate *Zbp1* knockout mouse. For detection of *Zbp1* ^−/−^ mice, one band with 315 bp was amplified using primer forward: 5’‐GGAGGATTGCTATGAGTTCCAGG‐3’ and reverse: 5’‐CTCTGGGTAGCTGATTCTTCCTCT‐3’. Wild‐type (WT) mice of 4‐8 weeks old were purchased from the Model Animal Research Center of Nanjing University. For pristane‐induced lupus‐like mouse model, 8 weeks old *Zbp1* ^−/−^ and WT mice were intraperitoneally injected with 500 µL pristane (Sigma, P9622) per mouse. Eight months after the pristane injection, the mice were sacrificed, and spleen, kidney, and serum samples were collected. All mice were maintained under specific pathogen‐free (SPF) conditions at the Center for New Drug Safety Evaluation and Research, China Pharmaceutical University. All mice used in this study were in C57BL/6J background. All animal experiments were performed in accordance with the National Institutes of Health Guide for the Care and Use of Laboratory Animals, with the approval of the Center for New Drug Safety Evaluation and Research, China Pharmaceutical University (202305‐003).

### 5.3 Cells

The mouse embryonic fibroblast (MEF) cells (ATCC Cat#SCRC‐1008) were purchased from the American Type Culture Collection (ATCC). Mouse bone marrow‐derived macrophages (BMDMs) were prepared as described previously [68]. For cell culture, MEFs were cultured in Dulbecco's modified Eagle's medium (DMEM) supplemented with 1% penicillin‐streptomycin (Invitrogen) and 10% fetal bovine serum (FBS) (Gibco).

### 5.4 Peripheral Blood Mononuclear Cell (PBMC) Isolation

The whole blood was collected from SLE patients and healthy donors, from which PBMCs were further isolated using Sepmate tubes (Stem cell Technologies) with Ficoll‐plaque (GE Healthcare Lifesciences). The isolated PBMCs were stored at ‐80°C before being used in experiments.

### 5.5 The STING Antagonist Treatment Experiment in BMDMs of *Trex1* ^−/−^ Mice

To inhibit the cGAS‐STING pathway in BMDMs, BMDMs from *Trex1^−/−^* mice were treated with SN‐011 (500 nm), H151 (500 nM) or DMSO for 12 h, and the corresponding expressing changes were assessed using RNA‐Seq experiments.

### 5.6 The STING Antagonist Administration Experiment in *Trex1* ^−/−^Mice

To pharmacologically inhibit the cGAS‐STING pathway in mice, *Trex1* ^−/−^ mice (1‐month old) were injected intraperitoneally with STING antagonist SN‐011 (10 mg/kg) or vehicle (1% Tween 80 in PBS) daily for two weeks, mice were then euthanized, and multiple tissues were collected for RNA‐Seq experiments, hematoxylin and eosin staining experiments, and immunohistochemistry experiments.

### 5.7 The cGAS Agonist Transfection Experiment

The transfection experiments of the cGAS‐specific agonist G3‐YSD (InvivoGen) and the corresponding control named G3‐YSD control (InvivoGen) were performed using Lipofectamine 2000 (Invitrogen) according to the manufacturer's instructions. In Brief, we conducted the transfection experiments using G3‐YSD (3 µg) and G3‐YSD control, and then collected total RNAs at 2, 4, 6, 8, 10, and 12 h after transfection in replicates. The RNA‐Seq experiments were further conducted to measure the corresponding transcriptome profiling. The G3‐YSD is a well‐established cGAS‐specific agonist, which flanks with guanosine trimers (G3) confers its cGAS agonist activity, while the G3‐YSD control differs from the G3‐YSD in the hairpin‐flanking nucleoside trimers, which flanks with cytidine trimers (C3) that abrogates cGAS activation [69].

### 5.8 The cGAMP Stimulation Experiment

For cGAMP stimulation, BMDMs in 12 well‐plates were incubated for 30 min at 37°C with cGAMP at different levels (0.125, 0.25, 0.6, 1, and 1.25 µg) in 500 µL of permeabilization buffer (50 mM HEPES, pH 7.0, 100 mm KCl, 3 mm MgCl2, 0.1 mm DTT, 85 mm sucrose, 0.2% BSA, 1 mm ATP, and 0.1 mm GTP) with 10 µg/mL digitonin (Sigma). Permeabilization buffer was then removed and replaced with DMEM or RPMI 1640 medium plus 10% FBS for 5.5 h before processing for RNA extraction and RNA‐Seq library construction.

### 5.9 The STING Antagonist Treatment Experiment in Human PBMCs

The extracted PBMCs of each SLE patient were separated into two parts, which were treated with SN‐011 (500 nm) or DMSO for 12 h, and the corresponding PBMCs were harvested, and RNA was extracted for RNA‐Seq experiments.

### 5.10 Hydroxychloroquine Treatment Experiment in Human PBMCs

For each healthy donor, the extracted PBMCs were separated into two parts, which were further incubated with hydroxychloroquine (20 µg, Sigma‐Aldrich) or DMSO for 24 h, respectively. After stimulation of HT‐DNA (4 µg, Sigma‐Aldrich) for 9 h, PBMCs were harvested, and RNA was extracted for RNA‐Seq experiments.

### 5.11 Histopathology

For hematoxylin and eosin (H&E) experiments of *Trex1* ^−/−^ mice, the collected tissues of *Trex1* ^−/−^ mice with STING antagonist SN‐011 treatment or PBS treatment were fixed in 4% (wt/vol) paraformaldehyde, paraffin‐embedded in paraffin, and sections were stained with hematoxylin and eosin. For H&E experiments of pristane‐induced lupus mice, the collected kidneys were fixed in 4% (wt/vol) paraformaldehyde, paraffin‐embedded in paraffin and sections were stained with hematoxylin and eosin. H&E‐stained kidney sections were scored in a blinded manner to determine a glomerular and interstitial inflammation score as described previous [70].

### 5.12 Immunohistochemistry Experiments

Immunohistochemistry (IHC) staining of mouse tissues was performed as described previously [36]. The antibodies used include rabbit anti‐MLKL (14993S, Cell Signaling), rabbit anti‐phospho‐MLKL (S358, ab187091, Abcam), mouse anti‐CXCL10 (SC‐347092, Santa Cruz Biotechnology), rabbit anti‐ZBP1 (PRS4401, Sigma‐Aldrich), mouse anti‐ISG15 (SC‐166755, Santa Cruz Biotechnology), and rabbit anti‐IFIT3 (15201‐1‐AP, Proteintech).

### 5.13 Flow Cytometry Analysis

Single‐cell suspensions of 1×10^6^ splenocytes were stained with flow antibodies, including anti‐CD4‐Brilliant Violet 605 (eBioscience, 406‐0042‐82), anti‐CD8‐FITC (BioLegend, 100706), anti‐CD69‐Brilliant Violet 421 (BioLegend, 104528), anti‐CD62L‐PE (eBioscience, 12‐0621‐81), anti‐CD44‐APC (BioLegend, 103012), anti‐CD45‐FITC (BioLegend, 103108), anti‐CD11b‐Brilliant Violet 605 (BioLegend, 101257), anti‐CD11c Brilliant Violet 421 (BioLegend, 117330), anti‐Ly6G‐PE (BioLegend, 127608), anti‐Ly6C‐APC (BioLegend, 128016), anti‐B220‐PE (BioLegend, 103208), anti‐CD27‐APC (BioLegend, 124212), anti‐CD138‐Brilliant Violet 421 (BioLegend, 142508). Flow cytometry was performed using CytoFLEX and analyzed using FlowJo.

### 5.14 Serum Autoantibody and Proteinuria Measurement

The serum sample was collected eight months after the pristane injection from *Zbp1* ^−/−^ and WT mice. The levels of autoantibodies (anti‐dsDNA) from sera (1:10 dilution) were measured by enzyme‐linked immunosorbent assay (CUSABIO, CSB‐E11194m) according to the manufacturer's instructions. An anti‐mouse albumin ELISA kit was used to measure urine (1:10 dilution) protein according to the manufacturer's instructions (BBI, D721120).

### 5.15 Immunoblotting Experiments

Immunoblotting experiments were performed as described previously [36]. The antibodies used include anti‐STING (13647, CST), anti‐TBK1 (ab40676, abcam), anti‐STAT1 (A19563, ABclonal), anti‐JNK (HA750018, HUABIO), anti‐JNK (HA750018, HUABIO), anti‐p38 (ET1702‐65, HUABIO), anti‐ACTB (A5441, Sigma‐Aldrich), anti‐Phospho‐STING (72971, CST), anti‐Phospho‐TBK1(5483, CST), anti‐Phospho‐STAT1 (AP0054, ABclonal), anti‐Phospho‐JNK (4668, CST), and anti‐Phospho‐p38 (4511, CST).

### 5.16 The RNA‐Seq Experiments and Gene Quantification Analysis

The total RNAs were extracted with the Trizol reagent (Invitrogen), subjected to polyA plus RNA enrichment using TruSeq RNA Library Preparation Kit v2 (Illumina), and further prepared into the cDNA library according to the Illumina strand‐specific RNA‐Seq instruction. Before library construction, the RNA integrity was estimated according to the RIN (RNA integrity number) value using Agilent 2100, and samples with the RIN value of no less than nine were retained for library construction. The constructed cDNA libraries were sequenced using Illumina HiSeq4000 in the 2 × 150 nt paired‐end layout. The raw RNA‐Seq data were processed using Trimmomatic to remove low‐quality reads and potential adaptor contaminations with the default parameter [71]. The resulting high‐quality RNA‐Seq data of human and mouse samples were mapped to the corresponding genomes using hisat2 with default parameters [72]. Only uniquely mapped read pairs were used for gene expression quantification using featurecounts [73].

### 5.17 The Identification of Gene Regulatory Modules of the cGAS‐STING Pathway

To identify major gene regulatory modules of cGAS‐STING pathway, we integrated the results of gene expression regression analysis, Markov Clustering, and protein‐protein interaction/association information of the STRING database. Specifically, we first conducted polynomial regression models to obtain the genes that were significantly associated with the cGAS‐STING signaling activation process, by adopting the method we developed previously [74, 75]. For each gene, by treating the cGAS‐STING pathway activation process as the predictor and gene expression abundance as the response, we selected the best regression model based on the “adjusted r^2^” criterion after applying all possible linear to cubic regression models. The significance of the selected regression model was estimated using the F‐test. The genes with an F‐test FDR value less than 10% were considered to be significantly associated with cGAS‐STING pathway activation, which were further classified into gene regulatory modules by applying Markov Clustering algorithm with default inflation parameters [76]. Only gene modules containing no less than 50 genes were retained. Finally, by incorporating the protein‐protein interaction/association information from STRING database [77], the gene regulatory modules of the cGAS‐STING pathway were obtained by filtering out the genes without interaction/association with other genes within a specific module. The functional enrichment of each gene module was estimated using David Bioinformatics (https://david.ncifcrf.gov/). M7core genes were defined and identified by intersecting the IRF2‐targeted M7 genes and M7 genes that were significantly induced after G3‐YSD stimulation.

### 5.18 Transcription Factor Binding Site Enrichment Analysis

The large‐scale transcription factor binding site (TFBS) data based on ChIP‐Seq experiments were downloaded from Gene Transcription Regulation Database (GTRD) [35]. For each gene, the region of 500 nt upstream and 500 nt downstream centered around the transcription start site was defined as its core promoter region, and the region of 1000 nt upstream and 1000 nt downstream centered around the transcription start site was defined as its proximal promoter region. The transcription factor regulation information on the core and proximal promoter region was obtained by intersecting the genomic loci of TFBS with the core and proximal promoter region, respectively. Fisher's exact test after multiple test correction was used to determine whether certain transcription factor has significantly more target genes in module 7 by taking the corresponding information of all expressed genes as the background (FDR < 0.05).

### 5.19 Case‐Control Diagnostic Performance Analysis

The AUROC analysis was used to estimate the diagnostic performance based on M7core genes or interferon‐stimulated genes. Two sets of interferon‐stimulated genes were used as the input to estimate the diagnostic performance. The first set of interferon‐stimulated genes was extracted from the database [78] by requiring an upregulation fold change more than 2. The second of interferon‐stimulated genes was extracted from the database [79] by requiring the FDR less than 5%.

### 5.20 In‐House Transcriptome Datasets

To elucidate the key gene signature of the cGAS‐STING pathway underlying lupus, we conducted systemic RNA‐Seq experiments and generated the following in‐house RNA‐Seq datasets, which include: The time‐course RNA‐Seq profiling after G3‐YSD stimulation in MEFs; The RNA‐Seq profiling in the BMDMs of WT and *Trex1* ^−/−^ lupus‐like mice with and without STING antagonist SN‐011 or H151 treatment; The RNA‐Seq profiling in BMDMs following stimulation with cGAMP of several levels; The RNA‐Seq profiling in multiorgan of *Trex1* ^−/−^ lupus‐like mice before and after STING antagonist SN‐011 administration; The RNA‐Seq profiling of the effect of HCQ in HT‐DNA treated human PBMCs; The RNA‐Seq profiling of PBMCs from 20 SLE patients and 6 healthy donors; The RNA‐Seq profiling of 27 SLE patients with and without the STING antagonist treatment; The RNA‐Seq profiling of kidneys from WT and *Zbp1* ^−/−^ pristane‐induced lupus‐like mice.

### 5.21 Public Transcriptome Datasets

Public blood transcriptome datasets from 9 independent SLE cohorts were downloaded from GEO and re‐analyzed, including P‐cohort1 (GSE80183), P‐cohort2 (GSE72509), P‐cohort3 (GSE49454), P‐cohort4 (GSE50772), P‐cohort5 (GSE39088), P‐cohort6 (GSE88884), P‐cohort7 (GSE110174), P‐cohort8 (GSE65391), P‐cohort9 (GSE110685). The clinical association between M7core and lymphopenia was analyzed based on the P‐cohort3 (GSE49454) including 157 blood samples of SLE patients. The clinical association between M7core and serologic and renal‐involved clinical manifestations was analyzed based on the P‐cohort8 (GSE65391), including 924 blood samples of SLE patients. Public RNA‐Seq profiles of all major immune cell‐types of PBMCs from SLE patients and healthy controls were downloaded from NBDC (JGAS000486) and re‐analyzed. The rheumatoid arthritis (RA) dataset, including 112 blood samples from RA patients was downloaded from GSE17755 and re‐analyzed. The transcriptome datasets from BMDMs and CD4^+^ T cells of WT, *Sting* ^−/−^, and serine 365‐to‐alanine (S365A) mutated mice with or without STING agonist DMXAA was downloaded from GSE149744 and re‐analyzed. The transcriptome datasets from heart of WT, *Trex1* ^−/−^, and *Sting* ^−/−^ *Trex1* ^−/−^ mice were downloaded from GSE59217 and re‐analyzed.

### 5.22 Statistical Analysis

Quantitative data are presented as mean ± standard error of the mean (S.E.M). Statistical analyses were performed using GraphPad Prism 8.0. For RNA‐Seq data, the negative binomial test from edgeR was used for differential expression analysis after TMM (trimmed mean of M‐values) normalization. Wilcoxon rank sum test was used for statistical analysis of two independent groups. ANOVA with post‐hoc Tukey HSD Test was used for statistical analysis of multiple groups. Multiple correction using Benjamini‐Hochberg false discovery rate (FDR) method was applied when multiple testing was performed. All statistical tests were two‐tailed. A *p*‐value of less than 0.05 was considered statistically significant for analyses without multiple testing. For analyses involving multiple testing, an FDR of less than 0.05 was considered statistically significant. Details regarding replicates are provided in the corresponding figure legends.

## Author Contributions

H.H., C.W., X.W. and H.Y. conceptualized this study; L.Z., Z.H., L.G., C.L., X.W., H.Y., JY.S. and M.M. performed experiments; M.A.L, J.S., J.H. and X.Z. performed data analysis, data visualization, data curation and technical support; H.H., L.Z., C.W., X.W. and Z.H. acquired funding for this study. H.H. wrote the original draft with contributions from all authors.

## Conflicts of Interest

The authors declare no conflicts of interest.

## Supporting information




**Supporting File 1**: advs74501‐sup‐0001‐SuppMat.pdf.

**Supporting File 1**: advs74501‐sup‐0002‐Tables.zip.

## Data Availability

All processed gene expression data from human and mouse samples have been provided in Supplementary Tables. The raw RNA‐Seq data from tissues, primary cells, and cell lines of mice were deposited in the Gene Expression Omnibus database (GEO) with the accession numbers GSE143826, GSE207458, GSE207859, GSE226594, GSE226672, and GSE311235. The raw RNA‐Seq data from SLE patients’ PBMCs are available upon reasonable request from the corresponding author, which are not publicly available due to privacy and ethical restrictions.
