# Supplementary material for: Integrative Analyses Identify a cGAS‐STING Pathway‐Driven Signature With Context‐Dependent Roles in Systemic Lupus Erythematosus
Source: Adv Sci (Weinh). 2026 Mar 3;13(25):e21560. doi: 10.1002/advs.202521560 (PMC13137784; doi:10.1002/advs.202521560)
Supplement: Supplementary file 1 — Supporting File 1: advs74501‐sup‐0001‐SuppMat.pdf. [file ADVS-13-e21560-s001.pdf]

Supplementary Figures

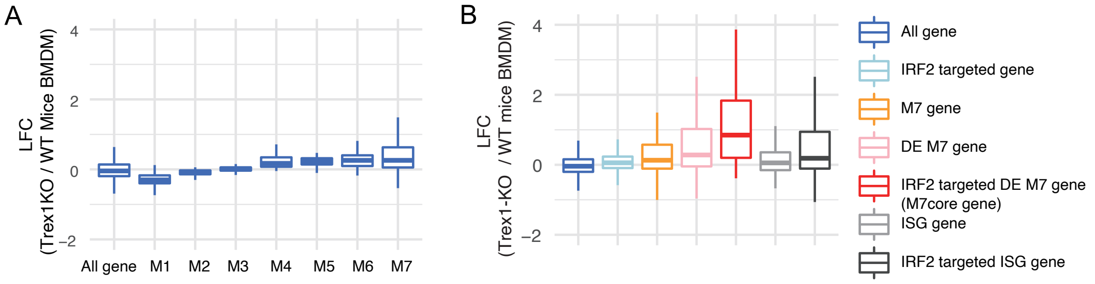

Figure S1: M7core exhibited the most substantial induction in the BMDMs of *Trex1*$^{-/-}$ lupus-like mice model. (A) The expression changes of seven gene modules of the cGAS-STING pathway in the BMDMs between *Trex1*$^{-/-}$ mice and wild-type (WT) mice. (B) The expression changes of M7core genes and the other six groups of genes (all genes, IRF2 targeted genes, M7 genes, and differentially expressed M7 genes, ISG genes, and IRF2 targeted ISG genes) in the BMDMs between *Trex1*$^{-/-}$ mice and WT mice.

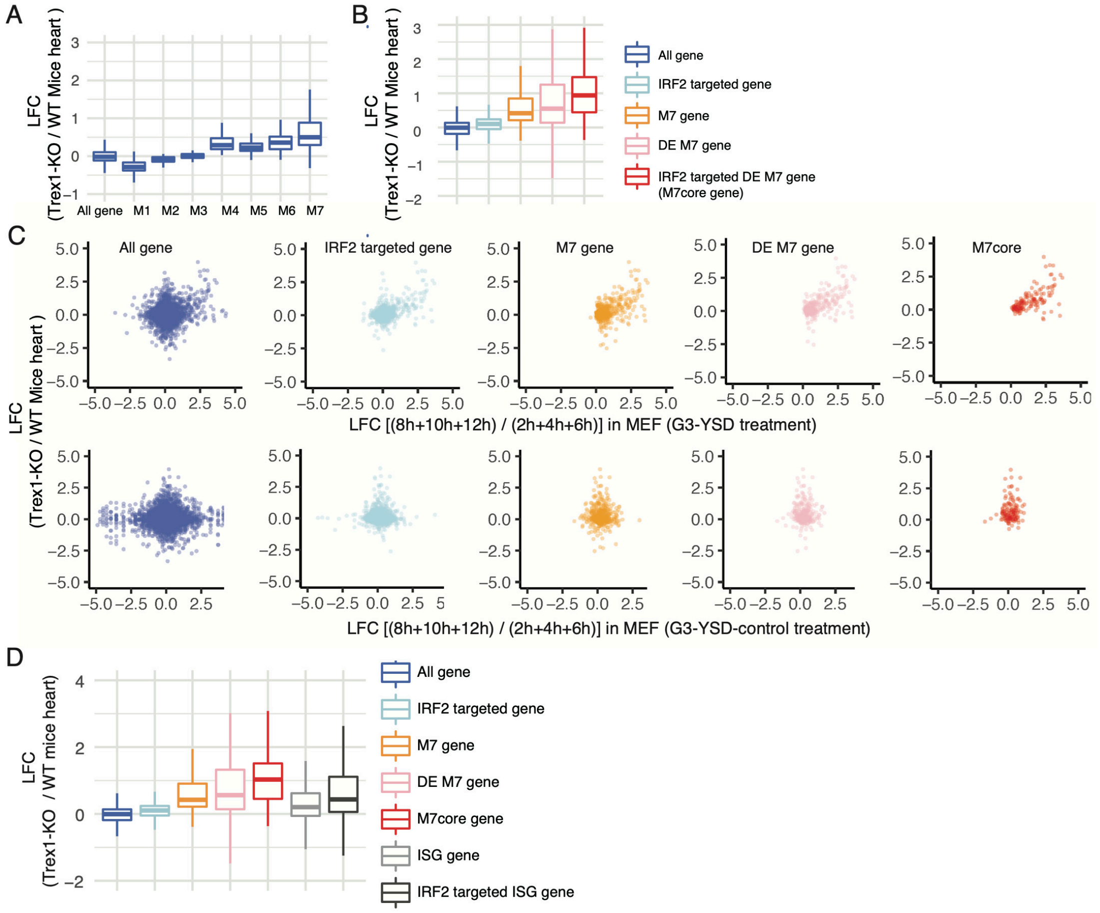

Figure S2: The M7core module exhibits the strongest induction in the heart of *Trex1*$^{-/-}$ lupus-like mice model. (A) The expression changes of seven gene modules of the cGAS-STING pathway in the heart between *Trex1*$^{-/-}$ mice and wild-type (WT) mice. (B) The expression changes of M7core genes and the other four groups of genes (all expressed genes, IRF2 targeted genes, M7 genes, and differentially expressed M7 genes) in the heart between *Trex1*$^{-/-}$ mice and WT mice. (C) The correlation between expression changes in the heart of *Trex1*$^{-/-}$ mice and expression changes after G3-YSD stimulation in MEFs for M7core genes and the other four groups of genes (upper row). The correlation between expression changes in the heart of *Trex1*$^{-/-}$ mice and expression changes after G3-YSD control sequence stimulation in MEFs for M7core genes and the other four groups of genes (bottom row). (D) The expression changes of M7core genes and the other six groups of genes (all genes, IRF2 targeted genes, M7 genes, and differentially expressed M7 genes, ISG genes, and IRF2 targeted ISG genes) in the heart between *Trex1*$^{-/-}$ mice and WT mice.

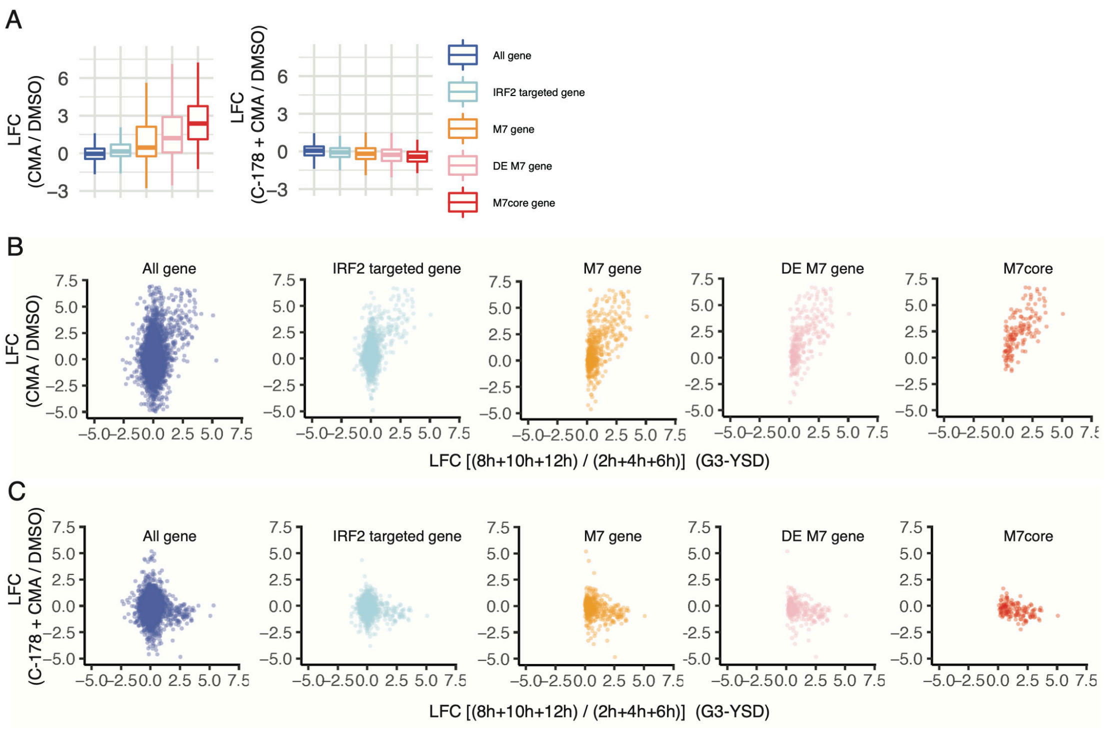

Figure S3: The expression of M7core genes were induced by STING agonist CMA and repressed by STING antagonist C-178 in BMDMs. (A) The expression changes of M7core genes and the other four groups of genes (all expressed genes, IRF2 targeted genes, M7 genes, and differentially expressed M7 genes) in the BMDMs after STING agonist CMA stimulation (left); The expression changes of M7core genes and the other four groups of genes (all expressed genes, IRF2 targeted genes, M7 genes, and differentially expressed M7 genes) in the STING antagonist C-178 treated BMDMs after STING agonist CMA stimulation (right). (B) The correlation between expression changes in CMA treated BMDMs and expression changes after G3-YSD stimulation in MEFs for M7core genes and the other four groups of genes. (C) The correlation between expression changes in CMA treated BMDMs and expression changes after G3-YSD control sequence stimulation in MEFs for M7core genes and the other four groups of genes.

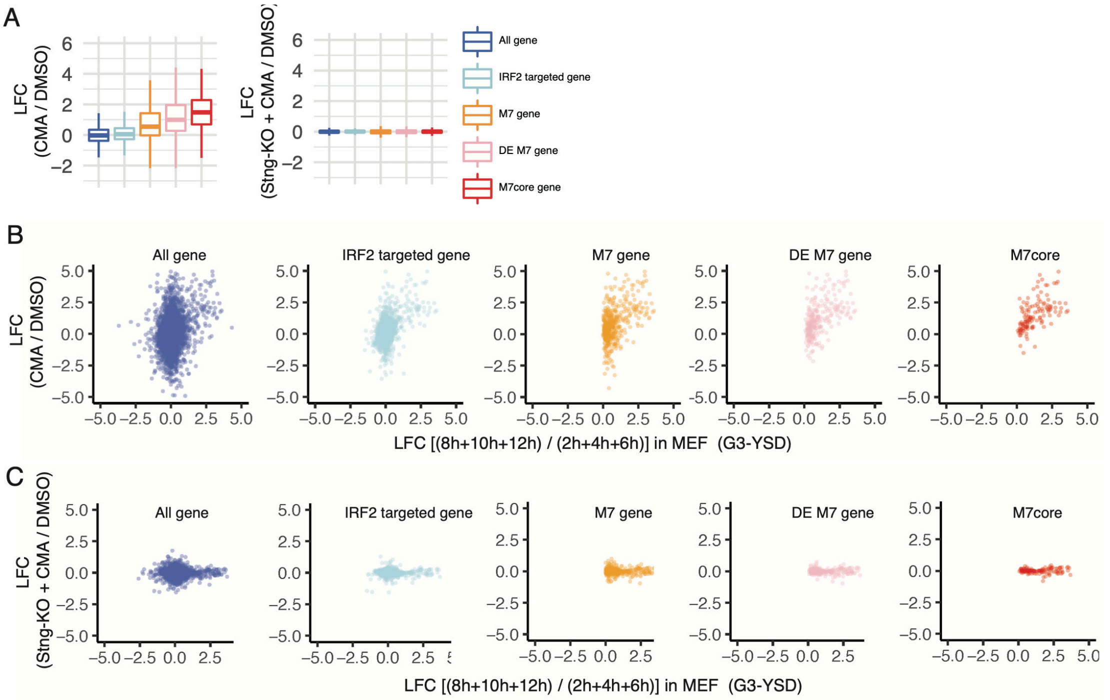

Figure S4: The expression of M7core genes were induced by STING agonist CMA and abolished in STING knock-out (KO) T cells. (A) The expression changes of M7core genes and the other four groups of genes (all expressed genes, IRF2 targeted genes, M7 genes, and differentially expressed M7 genes) in the` after STING agonist CMA stimulation (left); The expression changes of M7core genes and the other four groups of genes (all expressed genes, IRF2 targeted genes, M7 genes, and differentially expressed M7 genes) in the STING-KO T cells after STING agonist CMA stimulation (right). (B) The correlation between expression changes in CMA treated T cells and expression changes after G3-YSD stimulation in MEFs for M7core genes and the other four groups of genes. (C) The correlation between expression changes in CMA treated T cells and expression changes after G3-YSD control sequence stimulation in MEFs for M7core genes and the other four groups of genes.

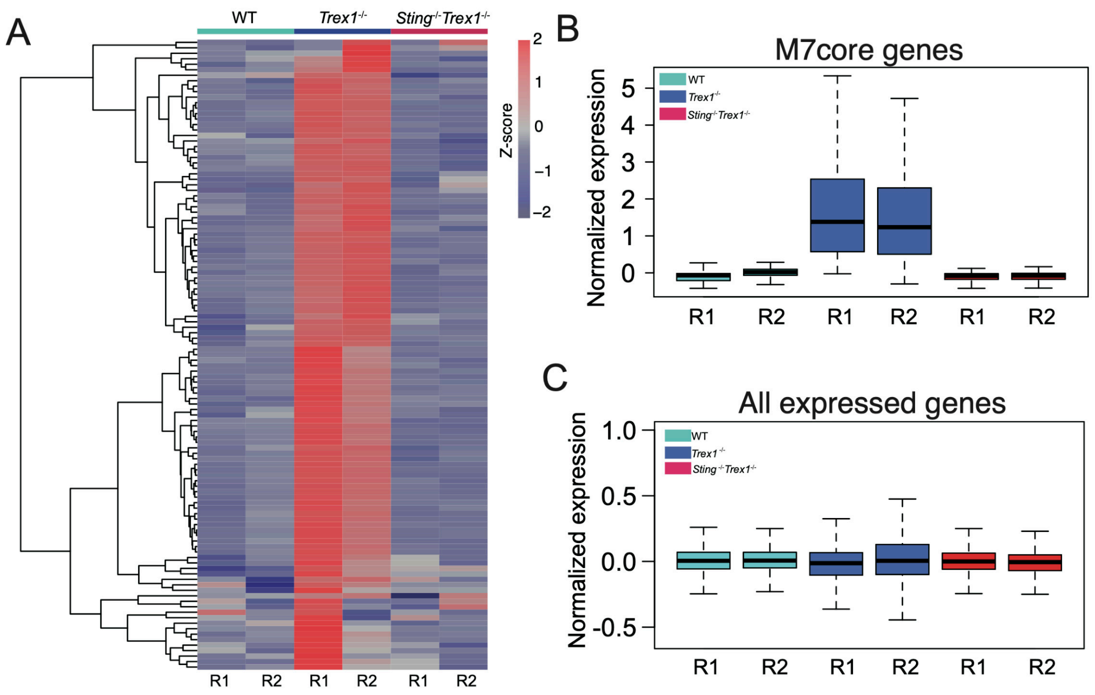

Figure S5: **Expression comparison of M7core genes in the heart of WT, *Trex1*$^{-/-}$ and *Sting*$^{-/-}$ *Trex1*$^{-/-}$ mice.** (A) heatmap showing normalized expression abundance (Z-score) of M7core genes in the heart of WT, *Trex1*$^{-/-}$ and *Sting*$^{-/-}$ *Trex1*$^{-/-}$ mice. (B-C) Boxplot showing the normalized expression abundance of M7core genes (B) and all expressed genes (C) in the heart of WT, *Trex1*$^{-/-}$ and *Sting*$^{-/-}$ *Trex1*$^{-/-}$ mice.

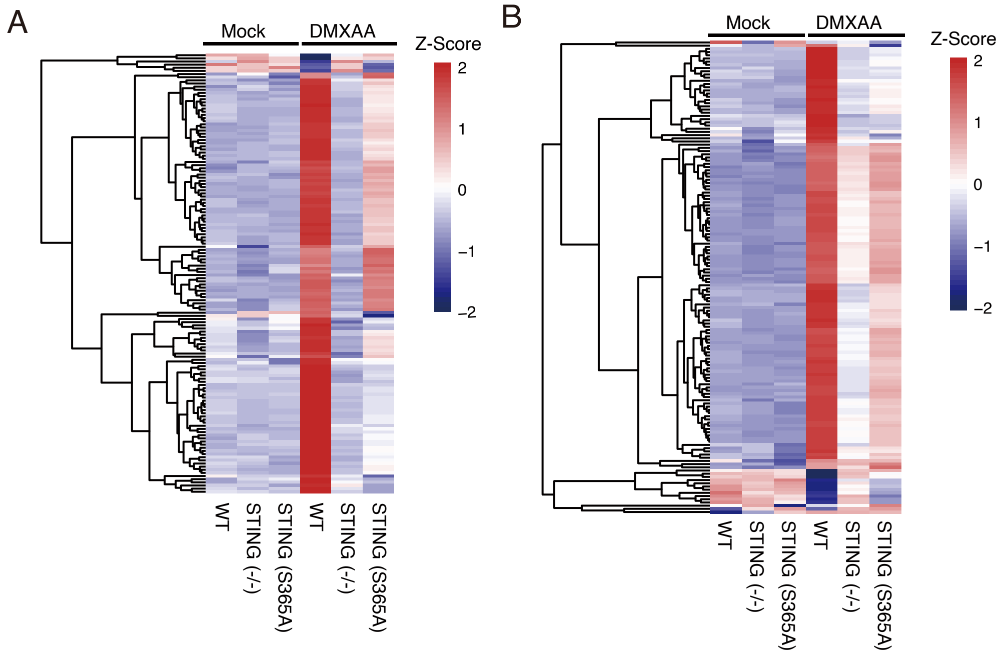

Figure S6: The expression abundance of M7core genes in the BMDMs and CD4+ T cells of WT, *Sting*$^{-/-}$, and serine 365-to-alanine (S365A) mutated mice with or without STING agonist DMXAA stimulation. (A) The heatmap of expression abundance of M7core genes in the BMDMs of WT, *Sting*$^{-/-}$, and serine 365-to-alanine (S365A) mutated mice with or without DMXAA stimulation. (B) The heatmap of expression abundance of M7core genes in the CD4^{+} T cells of WT, *Sting*$^{-/-}$, and serine 365-to-alanine (S365A) mutated mice with or without DMXAA treatment.

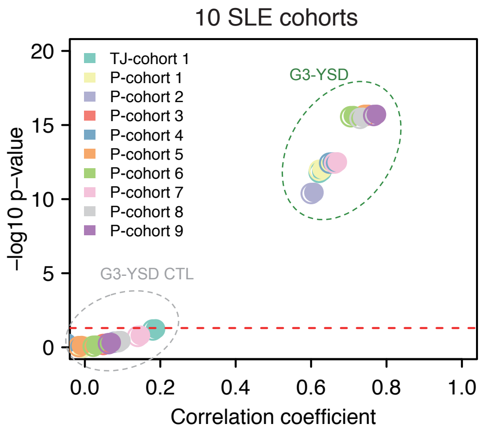

Figure S7: The correlation of the expression changes of M7core genes in SLE patients and in G3-YSD treated MEFs based on ten independent SLE datasets. The correlation between expression changes in SLE patients and expression changes after G3-YSD stimulation in MEFs for M7core genes across ten independent SLE cohorts (in green circle); The correlation between expression changes in SLE patients and expression changes after G3-YSD control stimulation in MEFs for M7core genes across ten independent SLE cohorts (in grey circle).

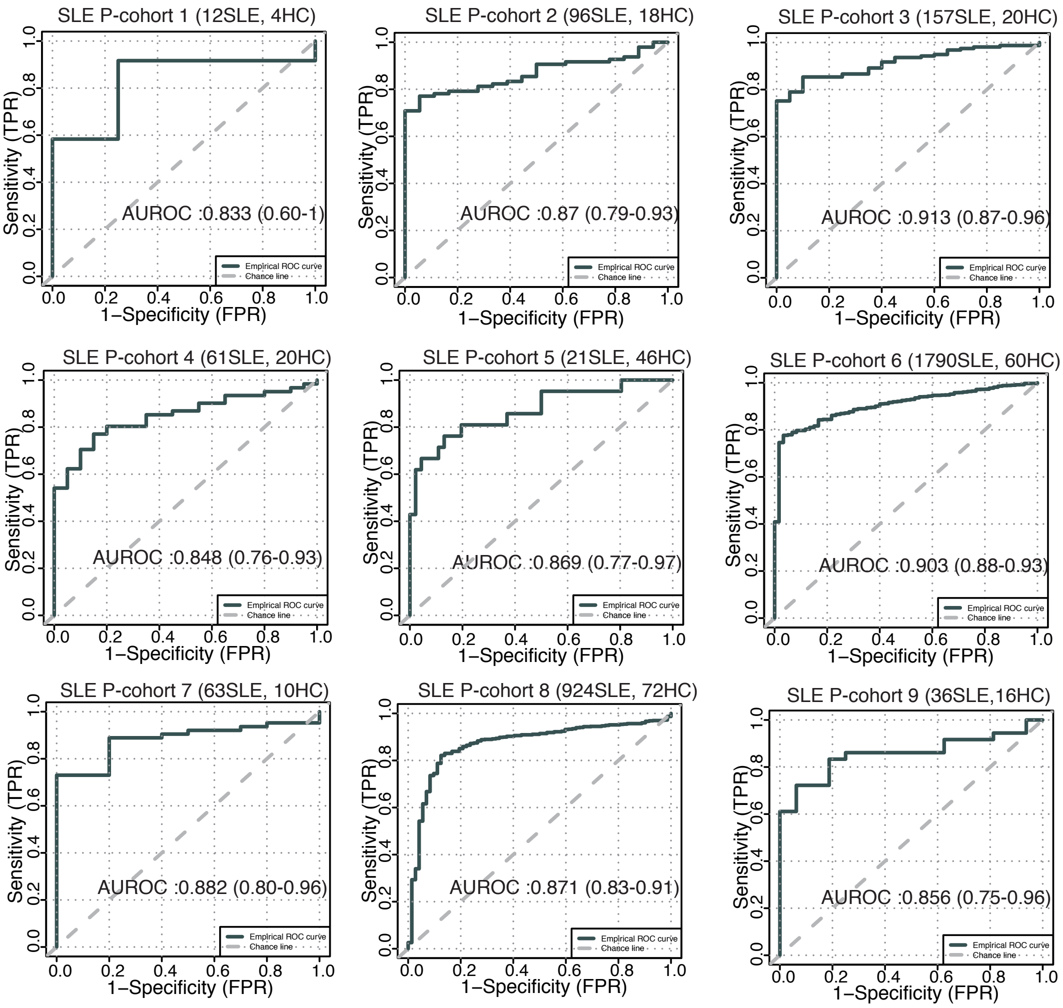

Figure S8: The Receiver Operating Characteristic (ROC) curve and Area Under Receiver Operating Characteristic Curve (AUROC) value based on M7core genes in nine independent SLE cohorts. In each panel, the ROC and AUROC value were calculated based on the expression of M7core genes in the blood of SLE patients (SLE) and Healthy donor controls (HC).

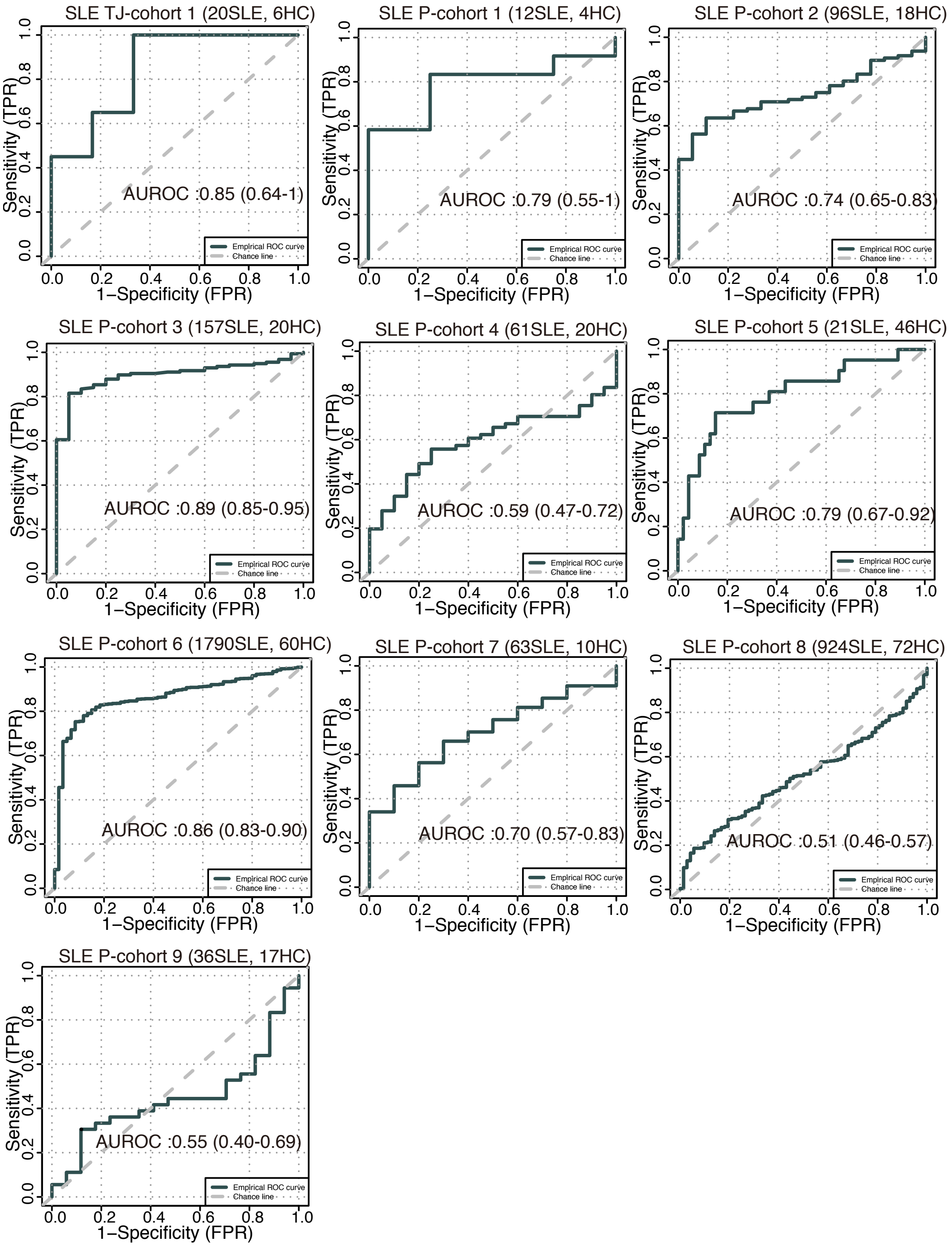

Figure S9: The Receiver Operating Characteristic (ROC) curve and Area Under Receiver Operating Characteristic Curve (AUROC) value based on ISG geneset1 in ten independent SLE cohorts. In each panel, the ROC and AUROC value were calculated based on the expression of ISG genes of geneset1 in the blood of SLE patients (SLE) and Healthy donor controls (HC).

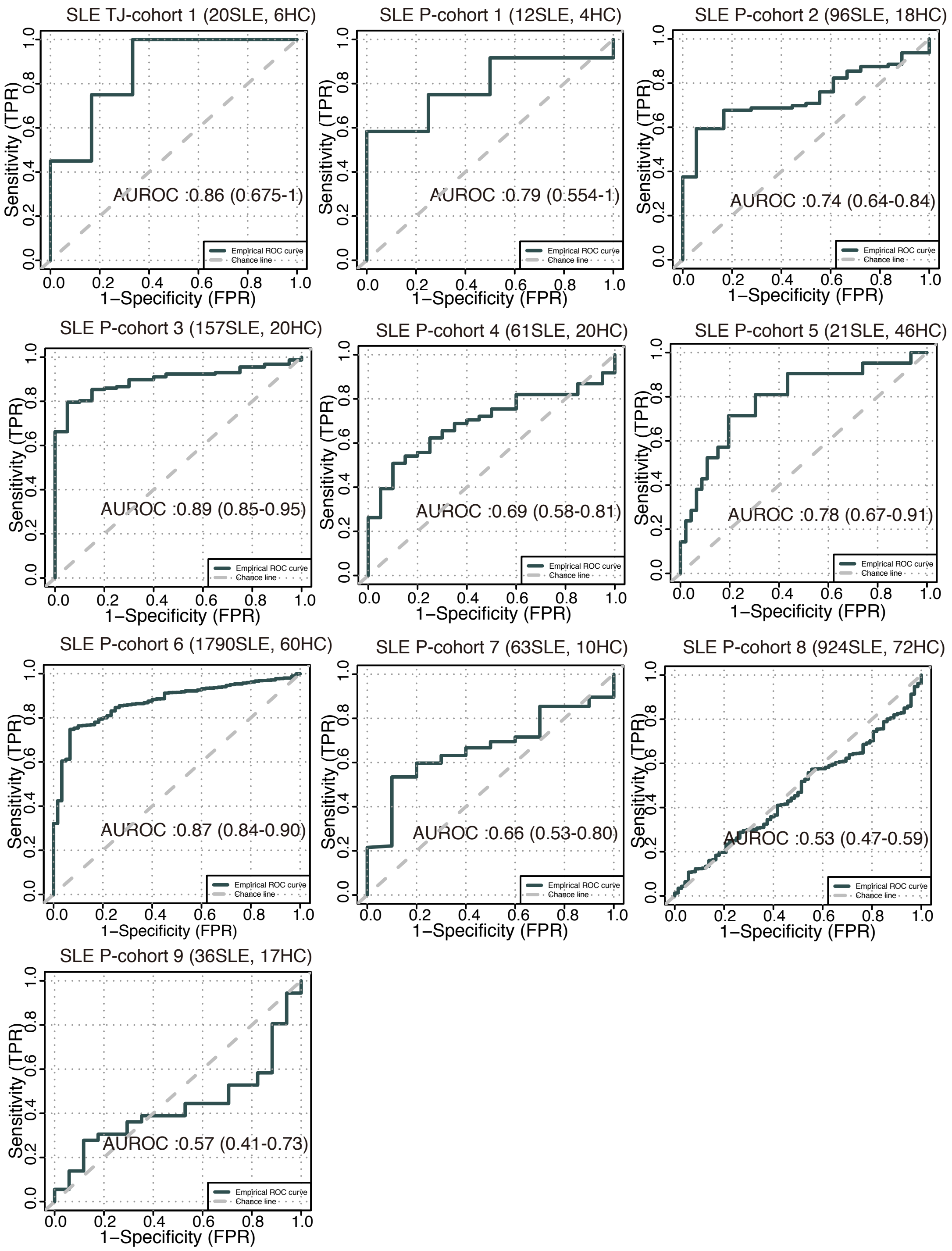

Figure S10: The Receiver Operating Characteristic (ROC) curve and Area Under Receiver Operating Characteristic Curve (AUROC) value based on ISG geneset2 in ten independent SLE cohorts. In each panel, the ROC and AUROC value were calculated based on the expression of ISG genes of geneset2 in the blood of SLE patients (SLE) and Healthy donor controls (HC).

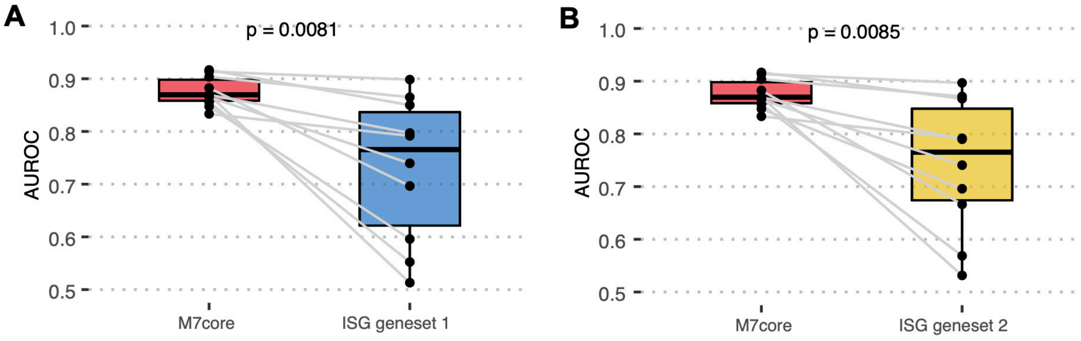

Figure S11: The comparison of Area Under Receiver Operating Characteristic Curve (AUROC) values calculated based on M7core genes and ISG genes in ten independent SLE cohorts. (A) The boxplot of AUROC values calculated based on M7core genes and ISG genes of geneset1 in 10 independent SLE cohorts. (B) The boxplot of AUROC values calculated based on M7core genes and ISG genes of geneset2 in 10 independent SLE cohorts. Statistical analysis was performed using two tailed paired Wilcoxon rank sum test for (A, B).

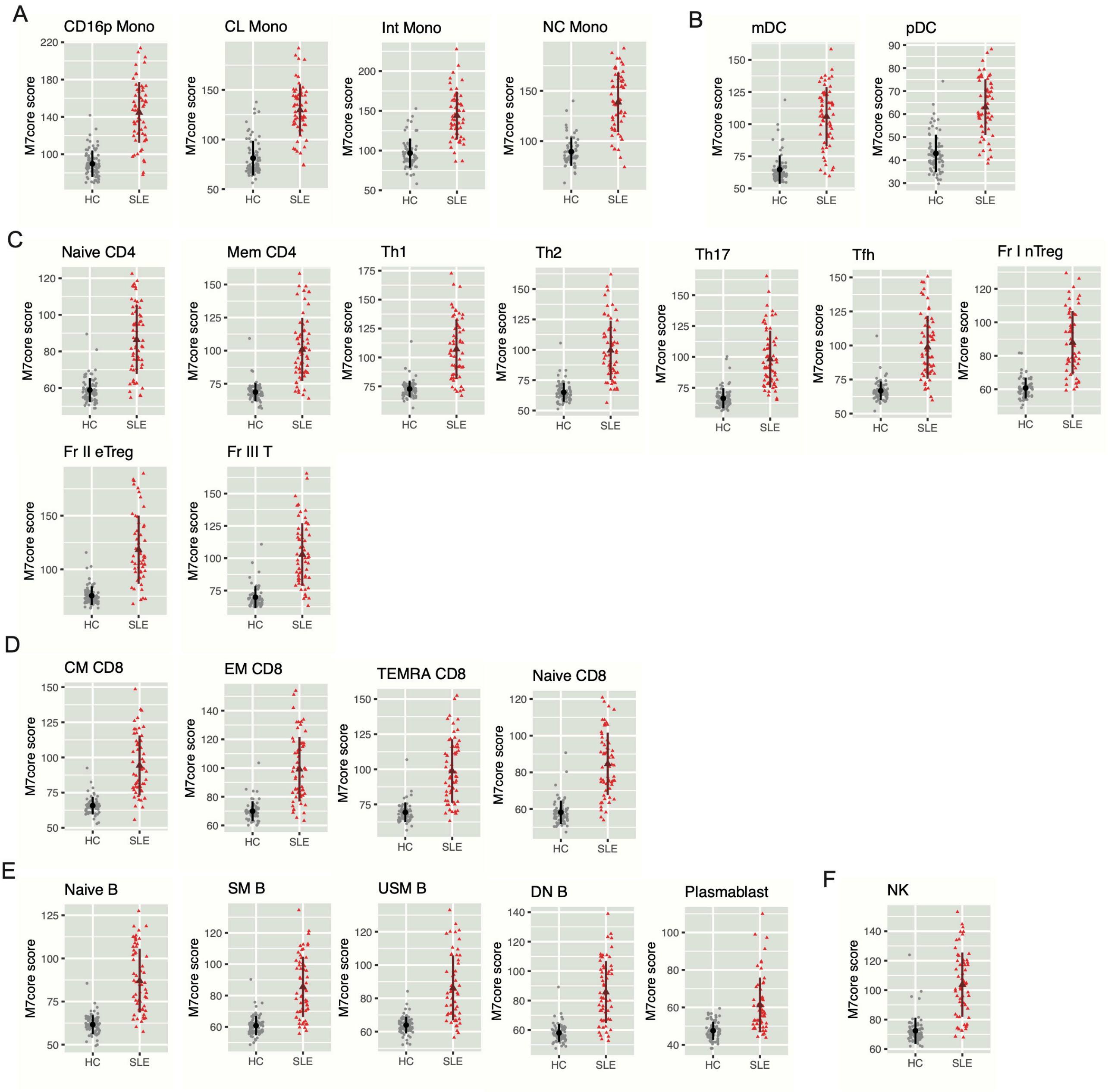

Figure S12: Comparison of M7core activity in 25 immune cells of PBMCs between SLE patents (SLE) and healthy controls (HC). (A-B) myeloid cell types across 4 subtypes of monocytes (A) and 2 subtypes of DC cells (B), and (C-F) lymphocyte types across 9 subtypes of $CD4^+$ T cells (c), 4 subtypes of $CD8^+$ T cells (D), 5 subtypes of B cells (E), and NK cells (F).

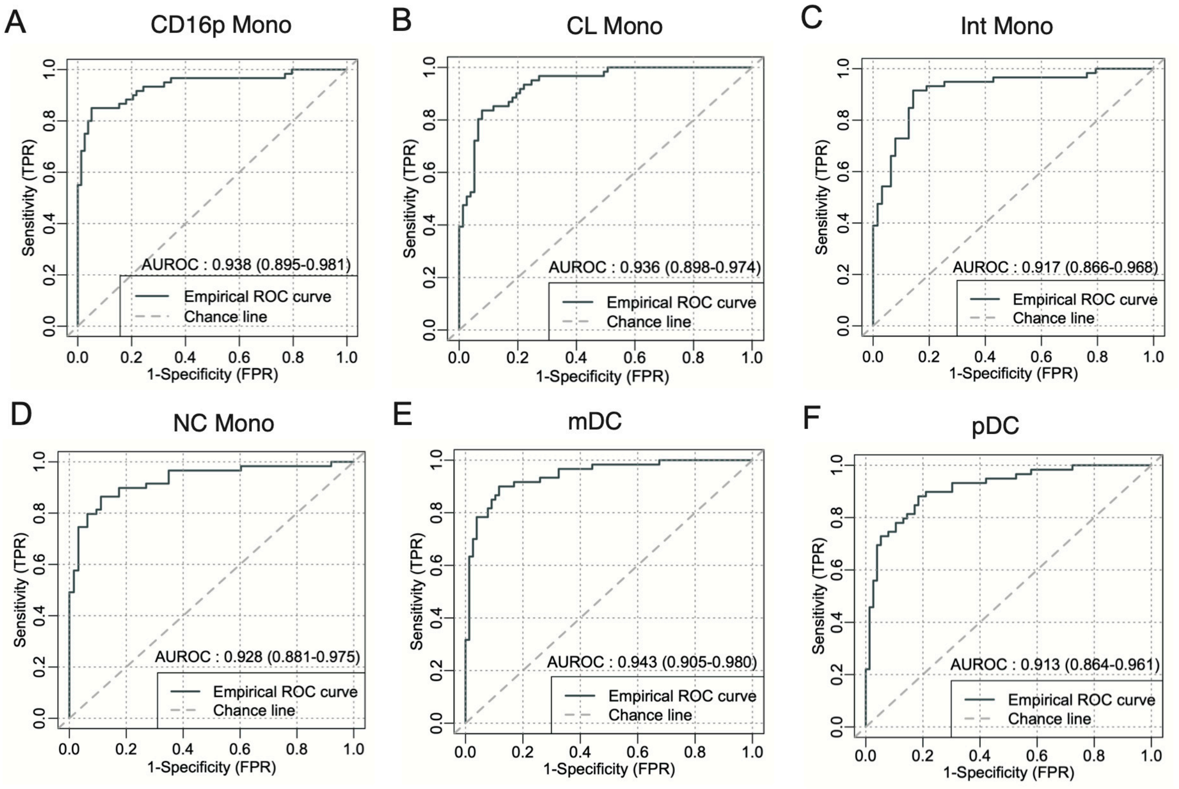

Figure S13: Receiver Operating Characteristic (ROC) curve and Area Under Receiver Operating Characteristic Curve (AUROC) value based on M7core activity across 6 subtypes of myeloid cells. In each panel, the ROC and AUROC value were calculated based on the expression of M7core genes in the SLE patients and Healthy controls.

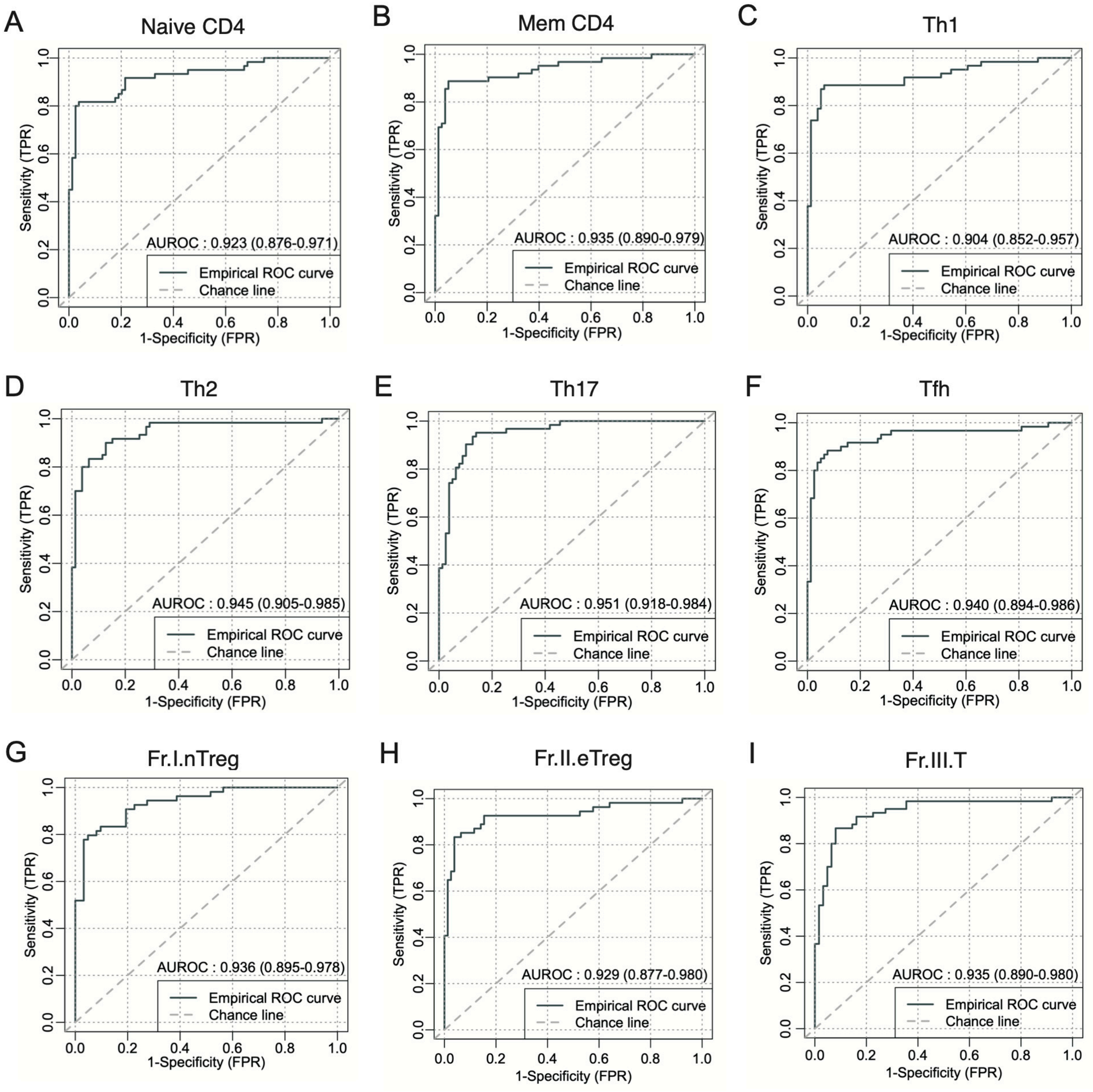

Figure S14: Receiver Operating Characteristic (ROC) curve and Area Under Receiver Operating Characteristic Curve (AUROC) value based on M7core activity across 9 subtypes of CD4^{+} T cells. In each panel, the ROC and AUROC value were calculated based on the expression of M7core genes in the SLE patients and Healthy controls.

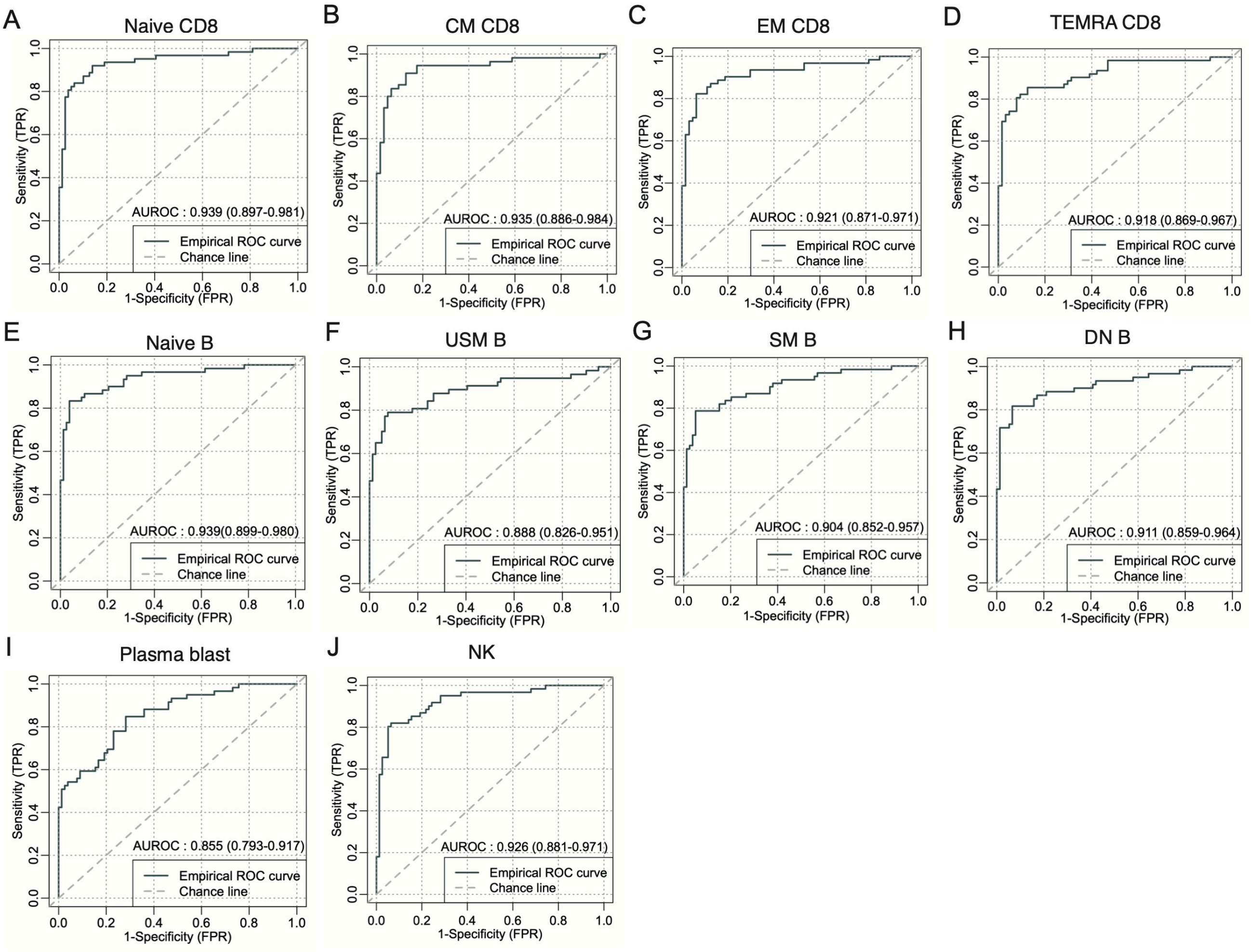

Figure S15: Receiver Operating Characteristic (ROC) curve and Area Under Receiver Operating Characteristic Curve (AUROC) value based on M7core activity across 4 subtypes of $CD8^{+}$ T cells (A-D), 5 subtypes of B cells (E-I), and NK cells (J). In each panel, the ROC and AUROC value were calculated based on the expression of M7core genes in the SLE patients and Healthy controls.

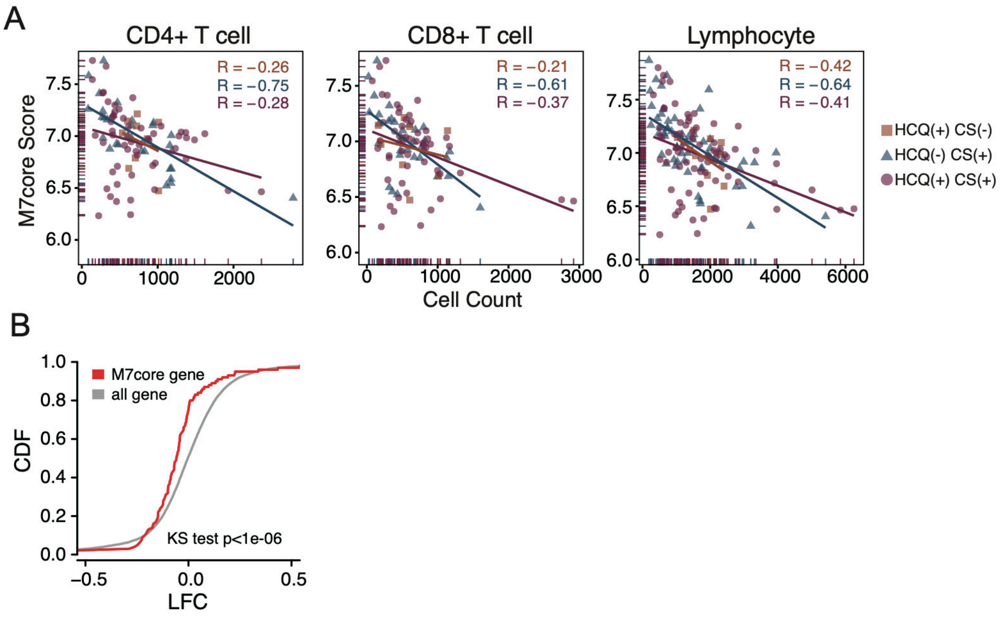

Figure S16: The comparison of the associations between M7core's activity and lymphopenia in the SLE patients after HCQ and/or CS treatments. (A) The association between the M7core's activity measured using M7core score and the counts of $CD4^+$ T cell, $CD8^+$ T cell, and lymphocyte in the SLE patients after HCQ and/or CS treatments, respectively. (B) The cumulative distribution function (CDF) plot of the expression changes of M7core genes in the HCQ treated PBMC after HT-DNA stimulation.

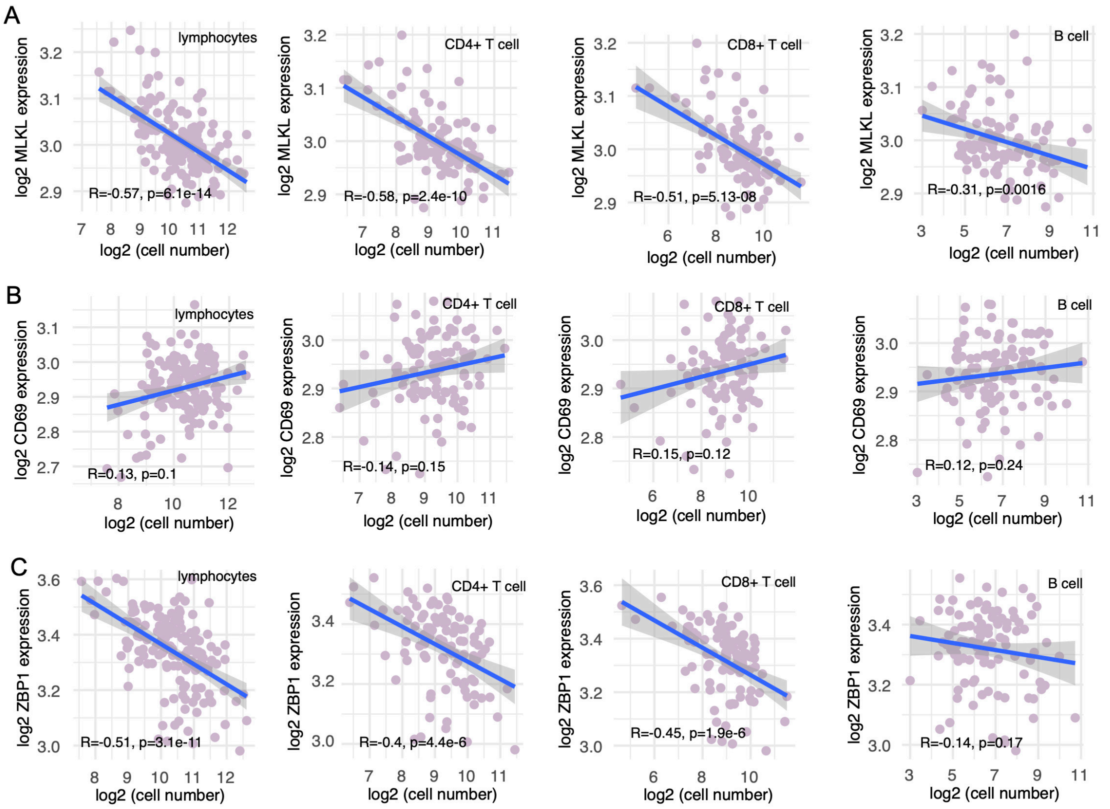

Figure S17: The associations between lymphopenia and the expression of MLKL, CD69, and ZBP1 in blood of the SLE patients. (A) The association between the expression abundance of MLKL and the counts of lymphocyte, $CD4^+$ T cell, $CD8^+$ T cell, and B cell in the SLE patients. (B) The association between the expression abundance of CD69 and the counts of lymphocyte, $CD4^+$ T cell, $CD8^+$ T cell, and B cell in the SLE patients. (C) The association between the expression abundance of ZBP1 and the counts of lymphocyte, $CD4^+$ T cell, $CD8^+$ T cell, and B cell in the SLE patients.

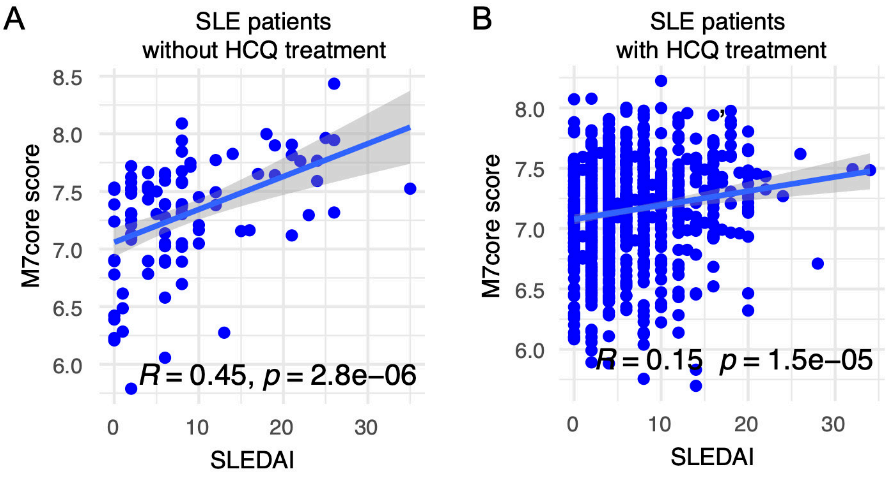

Figure S18: The correlation between M7core's activity and SLE Disease Activity Index (SLEDAI) for the SLE patients with and without HCQ treatment. (A) The correlation between M7core's activity measured using M7core score and SLE disease measured using SLEDAI in the SLE patients without HCQ treatment. (B) The correlation between M7core's activity measured using M7core score and SLE disease measured using SLEDAI in the SLE patients with HCQ treatment.

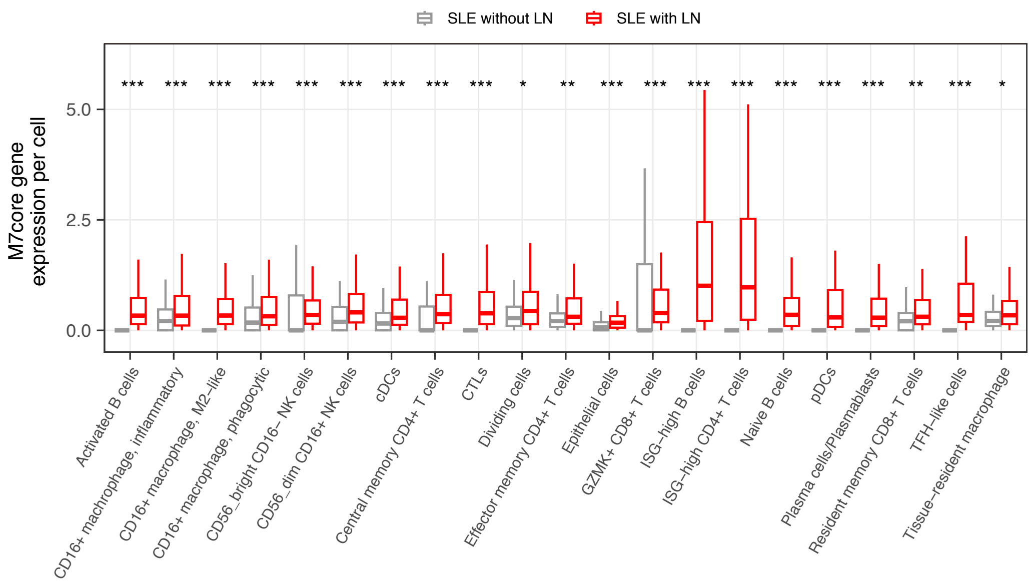

Figure S19: The comparison of expression abundance of M7core genes in the infiltrating leukocytes of the kidneys between SLE patients with lupus nephritis and SLE patients without lupus nephritis based on single cell sequencing. The abundance of M7core genes in each cell of the infiltrating leukocytes in the kidney was estimated using the sum of M7core genes due to the relatively low coverage of expressed genes of single cell sequencing. Statistical analysis was performed using ANOVA followed by Tukey's test. Statistical significance is represented by * $p<0.05$, ** $p<0.01$, *** $p<0.001$

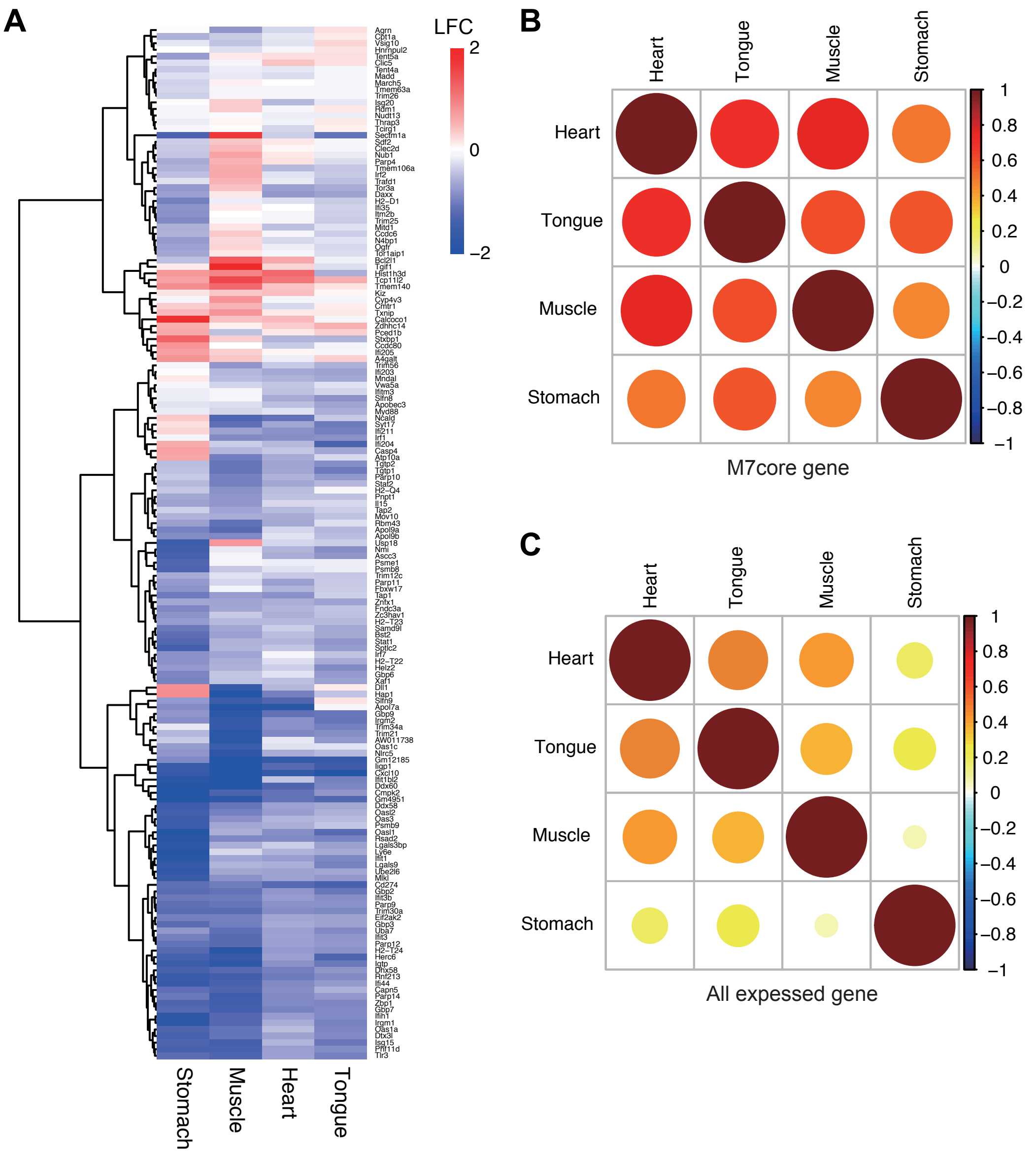

Figure S20: The expression changes of M7core genes in multiple affected tissues after administration of two-week STING antagonist SN-011 in *Trex1*$^{-/-}$ mice. (A) The heatmap of the expression changes of M7core genes in multiple affected tissues (Stomach, Muscle, Heart, and Tongue) after administration of two-week STING antagonist SN-011 in *Trex1*$^{-/-}$ mice. (B) The pairwise correlation of the expression changes of M7core genes in multiple affected tissues after administration of two-week STING antagonist SN-011 in *Trex1*$^{-/-}$ mice. (C) The pairwise correlation of the expression changes of all expressed genes in multiple affected tissues after administration of two-week STING antagonist SN-011 in *Trex1*$^{-/-}$ mice.

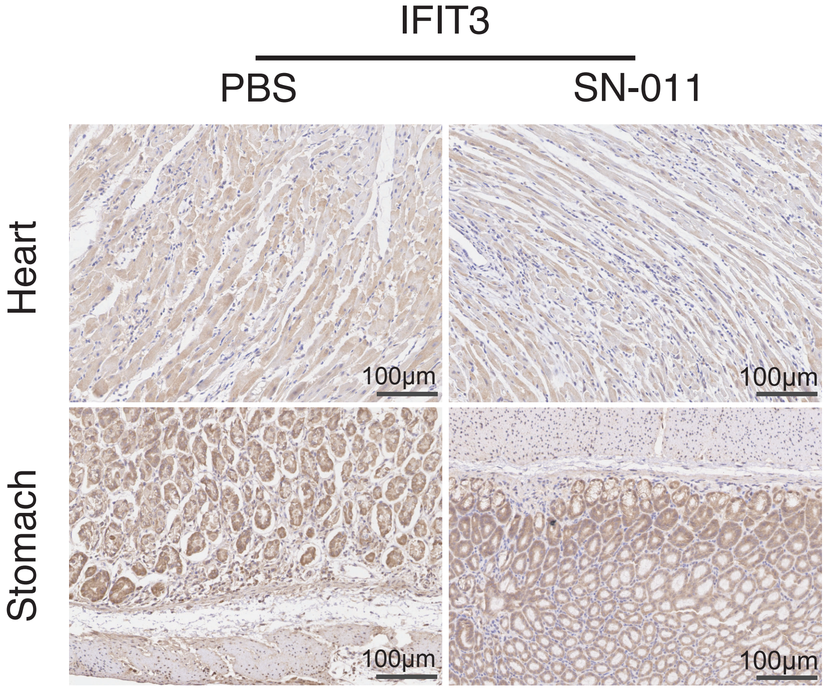

Figure S21: The representative immunohistochemistry (IHC) staining of IFIT3. Representative IHC staining of IFIT3 in the heart and stomach of *Trex1*$^{-/-}$ mice with two-week administration of STING antagonist SN-011 or PBS. Scale bar, 100 µm

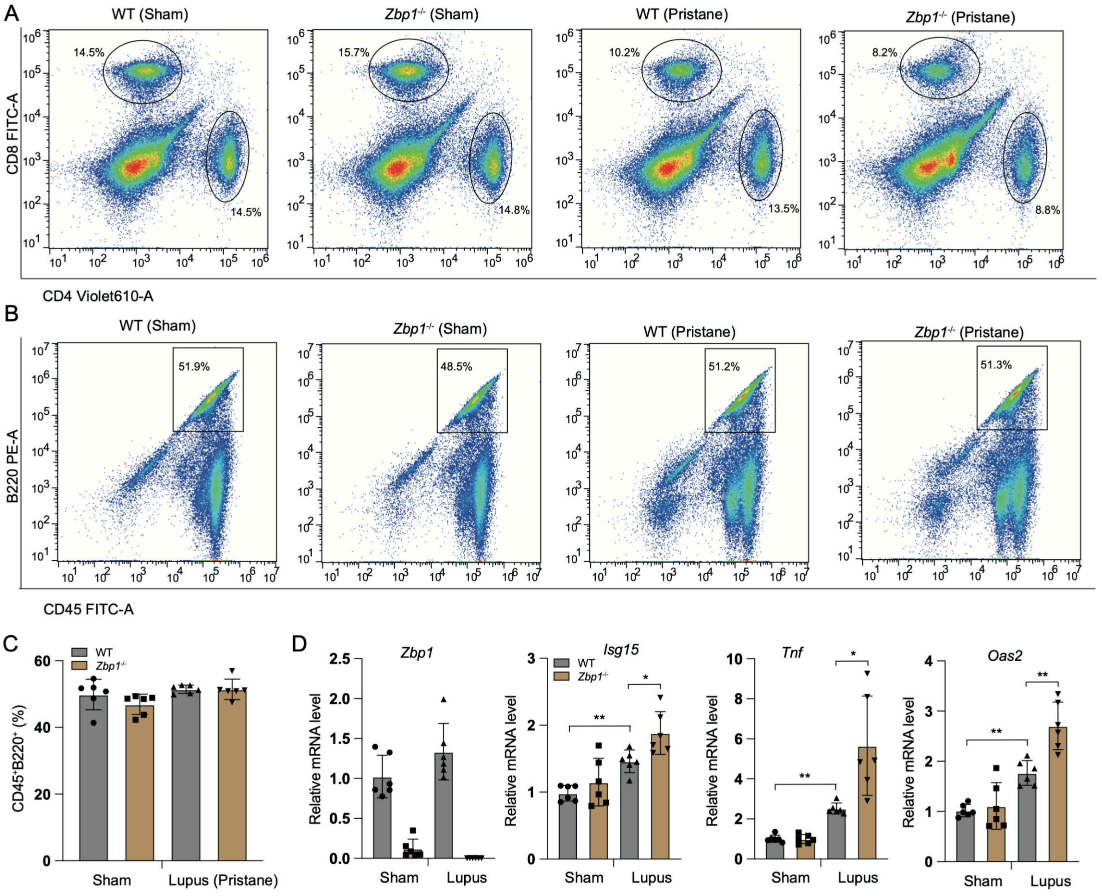

Figure S22: ZBP1 deficiency exacerbates autoimmune pathology in pristane-induced lupus-like mice. (A) Representative dot plots of CD4⁺ and CD8⁺ lymphocytes measured by flow cytometry. (B-C) The percentage and representative dot plots of Pan B cells (CD45⁺B220⁺) in the spleens of the experimental mice. (D) Gene expression of *Zbp1*, *Tnf*, *Isg15*, and *Oas2* in the kidney of the experimental mice was assessed by quantitative PCR (n=6, per group). All data in the statistical plots are shown as mean ± SD. Statistical analysis was performed using ANOVA followed by Tukey's test (C, D). Statistical significance is represented by *$p < 0.05$, **$p < 0.01$.

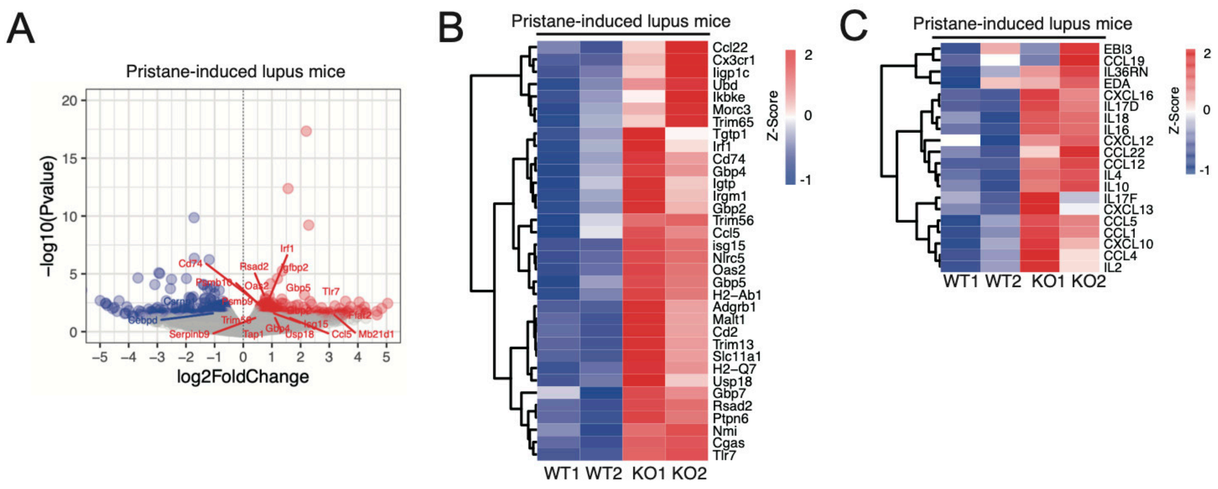

Figure S23: Significant upregulation of ISGs, genes relating to regulation of cytokines, and several cytokines in *Zbp1*-deficient kidneys in pristane-induced lupus-like mice. (A) Volcano plot of differentially upregulated (in red) and downregulated (in blue) genes in *Zbp1*$^{-/-}$ kidneys compared with WT counterpart under pristane-induced lupus-like model. The differentially upregulated and downregulated ISGs were labeled in red and blue, respectively. (B) Heatmap showing significantly induced genes relating to regulation of cytokines in *Zbp1*$^{-/-}$ kidneys compared with WT counterpart under pristane-induced lupus-like model. (C) Heatmap showing significantly induced cytokines in *Zbp1*$^{-/-}$ kidneys compared with WT counterpart under pristane-induced lupus-like model.

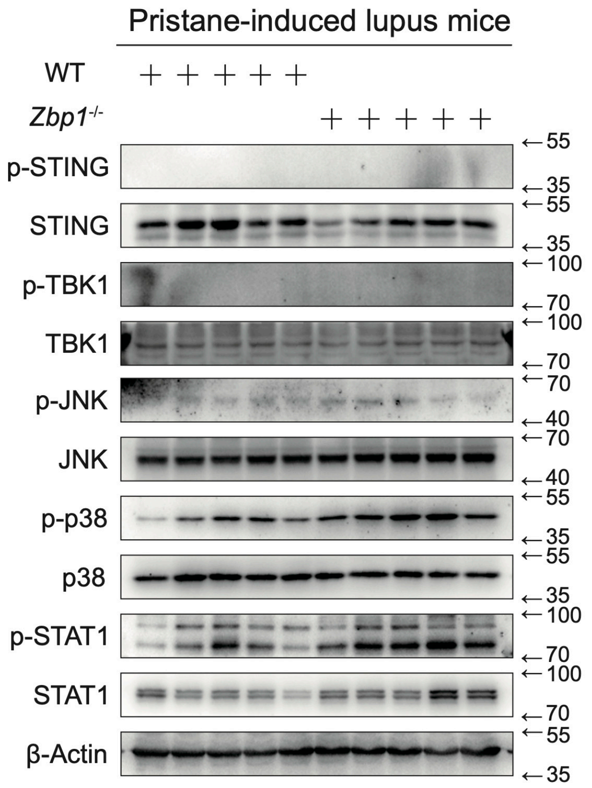

Figure S24: Substantial activation of p38 (p-p38) and STAT1 (p-STAT1) in *Zbp1*$^{-/-}$ kidneys of pristane-induced lupus-like mice. Western blot results of STING, TBK1, JNK, p38, STAT1, phosphorylated STING (p-STING), phosphorylated TBK1 (p-TBK1), phosphorylated JNK (p-JNK), phosphorylated p38 (p-p38), and phosphorylated STAT1 (p-STAT1) in WT and *Zbp1*$^{-/-}$ kidneys of pristane-induced lupus-like mice (n=5, per group).
